# A computer-aided method for controlling chemical resistance of drugs using RRKM theory in the liquid phase

**DOI:** 10.1038/s41598-021-01751-z

**Published:** 2021-11-26

**Authors:** Hamed Douroudgari, Morteza Vahedpour

**Affiliations:** Department of Chemistry, University of Zanjan, PO Box 38791-45371, Zanjan, Iran

**Keywords:** Molecular biophysics, Biophysical chemistry, Chemistry, Theoretical chemistry

## Abstract

The chemical resistance of drugs against any change in their composition and studying the rate of multiwell-multichannel reactions in the liquid phase, respectively, are the important challenges of pharmacology and chemistry. In this article, we investigate two challenges together through studying drug stability against its unimolecular reactions in the liquid phase. Accordingly, multiwell-multichannel reactions based on 1,4-H shifts are designed for simplified drugs such as 3-hydroxyl-1H-pyrrol-2(5H)-one, 3-hydroxyfuran-2(5H)-one, and 3-hydroxythiophen-2(5H)-one. After that, the reverse and forward rate constants are calculated by using the Rice Ramsperger Kassel Marcus theory (RRKM) and Eckart tunneling correction over the 298–360 K temperature range. Eventually, using the obtained rate constants, we can judge drug resistance versus structural changes. To attain the goals, the potential energy surfaces of all reactions are computed by the complete basis set-quadratic Becke3 composite method, CBS-QB3, and the high-performance meta hybrid density functional method, M06-2X, along with the universal Solvation Model based on solute electron Density, SMD, due to providing more precise and efficient results for the barrier heights and thermodynamic studies. To find the main reaction pathway of the intramolecular 1,4-H shifts in the target molecules, all possible reaction pathways are considered mechanistically in the liquid phase. Also, the direct dynamics calculations that carry out by RRKM theory on the modeled pathways are used to distinguish the main reaction pathway. As the main finding of this research, the results of quantum chemical calculations accompanied by the RRKM/Eckart rate constants are used to predict the stability of drugs. This study proposes a new way to examine drug stability by the computer-aided reaction design of target drugs. Our results show that 3-hydroxyfuran-2(5H)-one based drugs are the most stable and 3-hydroxythiophen-2(5H)-one based drugs are more stable than 3-hydroxy-1H-pyrrol-2 (5H)-one based drugs in water solution.

## Introduction

Heterocyclic compounds include several carbon atoms that have been imposed in a ring (carbocyclic compounds), and also some carbon atoms have been replaced by nitrogen or oxygen and or sulfur as a hetero atom^1–4^. As is well-known, the five-membered heteroaromatic rings containing oxygen and sulfur atoms, furan and thiophene, are frequently used as building blocks in many drugs^5–7^. Aminoquinoline derivatives that are based on thiophene and furan rings have been found to be potent inhibitors of polymerization of b-hematin and also is antimalarials^8^. Furan ring plays an important role in the neoclerodane diterpene salvinorina compound. However, after the substitution of some alkyl groups to α carbon of furan, it converts to a potent agonist κ-opioid receptor^9^. Two highly reactive thiophene-based metabolites are thiophene epoxides and thiophene S-oxides. In drug-induced hepatotoxicity, these reactive electrophilic thiophene-based metabolites are key compounds^10^.

In recent decades, nitrogen-based heterocyclic compounds have been considered seriously by many researchers as useful subunits and intermediates in the synthesis of biological or pharmaceutical compounds^11–16^. As an example, we can refer to the Indole scaffold. It is a planar bicyclic molecule that contains a pyrrol ring and is a main structural subunit in discovering new drugs^17^.

**Scheme 1:**
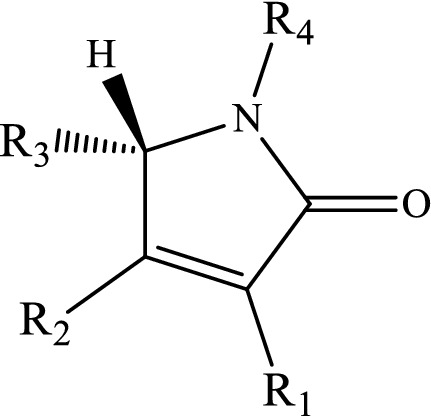
General molecular structure of pyrrolidinone-based compounds (R1–R4 are different functional groups see Refs.^18,19^).

Another very important example is pyrrolidinone-based compounds in which the pyrrol ring is their central framework (see Scheme 1). Nowadays, these compounds are contributed to human life^18,19^. They exist in many pharmaceutical drugs and natural products such as (−)-Azasprine, Lactacystin, tobacco, and Salinosporamide and are a metabolite of nicotine and many biological activities^19–23^.

Recently, the handling of expired drugs due to increased risk of toxicity is known as a global canker^24^. On the other hand, the expired drugs and unused ones which are stored at home are pharmaceutical waste^25^. The influence of pharmaceutical waste is multifaceted on different aspects of human life. From an economic point of view, a massive loss of financial resources is necessary for pharmaceutical waste, which inflicts an ecological economic on involved countries^26^. So, designing drugs with a longer lifetime is accounted as a significant challenge.

Knowledge of possible reactions that cause a change in the structure of available drugs could open new ways for pharma companies to increase the lifetime of newly produced ones. In this research, we demonstrated that mechanistic and kinetic evaluations will lead us to the intended goal. This study also follows this question ‘‘which drug has a longer lifetime of usage?’’ In the sequel, we look for the response to this question by modeling possible computer-aided reactions for the mechanistic and kinetic study. As an example, in this article, we study the mechanism and rate constants of the 1,4-H shifts in 3-hydroxy-1H-pyrrol-2(5H)-one, 3-hydroxy-1H-pyrrol-2(5H)-one, and 3-hydroxy-1H-pyrrol-2(5H)-one compounds as a simple representation of drugs. One of the aims of this study is to predict the stability of drugs by obtaining more accurate rate constants in different conditions. For this purpose, multiwell-multichannel reactions associated with the mentioned unimolecular reaction are explored by the validated methods such as CBS-QB3, and M06-2X. Also, our study proves that the CBS-QB3 and M06-2X methods have sufficient accuracy to investigate the kinetics and thermodynamics of drugs due to having the results close to each other. We also show that 1,4-hydrogen shifts in pyrrolidinone-based compounds occur via two distinct paths. The first occurs by jumping over carbon atoms and the second happens through jumping over the heteroatom. These reaction pathways are designed in the solution phase. However, for the dynamics calculations, the RRKM theory along with the Eckart tunneling factor is implemented to calculate the rate constants of considered multiwell-multichannel potential energy surfaces (PESs). By surveying the literature, it can be concluded that this is the first study for the calculation of intramolecular 1,4-H shifts in drugs using high-level quantum chemical calculations. On the other hand, to the best of our knowledge, up to now, less research attention has been paid to determine the rate of complex reactions containing different wells and channels in liquid comprehensively. Implying a reliable solvation model like the SMD solvation model could play a crucial role in determining rates of multiwell-multichannel unimolecular reactions in the liquid phase.

### Computational details

#### Inclusion of solvent into the calculations

In the theoretical study of the liquid phase chemical reactions, the crucial strategy is to insert the solvent in calculations in which the reaction happens, especially when we use quantum chemical methods. Two main approaches have been accepted in the literature. In the first approach, large numbers of solvent molecules explicitly are arranged around the reacting molecules, and then the target calculations are carried out. This approach, together with quantum chemistry methods, has a high computational cost. So, it does get much less attention. In the second, instead, chemists use the solvation models^27^. These models apply the effect of solvent implicitly. The well-known implicit solvation model, which has received the most attention in the last decade, is the universal solvation model based on solute electron density, SMD^28^. It is a continuum solvation model. For solute–solvent interaction, it uses a continuum description of the solvent together with the full solute electron density. A large number of solvation free energies data (2821) have been used to parameterize this model. Thus, it can apply to different types of solute (charged or uncharged) in any solvent (liquid medium) with more accurate results than the other models. Therefore, for all reactions in liquid, we used the SMD solvation model to simulate the solvent (water) molecules’ effects on the solute molecules (drugs) implicitly.

#### Electronic structure calculation methods

All stationary points such as reactants (R), intermediates (IN), transition states (TS), and products (p) were fully optimized in the liquid phase. For employing better convergence behavior and remarkably higher computational efficiency, we used the M06-2X^29^ method as a validated meta hybrid density functional approach for geometry optimization using two suitable augmented triple zeta basis sets. The first basis set was Pople type, 6-311 + g(2df,2p), and the second was Dunning type, Jun-cc-pVTZ (or Jun-cc-pV(T+d)Z for S atom)^30^. Other reasons to use the M06-2X method with two basis sets are: (a) examining the sensitivity of reaction components to different basis sets and (b) having successful results in the simulations of kinetic, thermochemistry, and noncovalent interactions among solute and solvent molecules^31^. The frequency calculation was carried out at the M06-2X/6-311+g(2df,2p) and M06-2X/Jun-cc-pVTZ (and M06-2X/Jun-cc-pV(T+d)Z for S atom) levels of theory for all species of the liquid phase reactions. We used the calculated frequencies to verify the nature of stationary points as true minima, such as reactant (R), intermediate (IN), and product (P) and true first-order saddle points (TSs), and also to gain zero-point energies (ZPEs), partition functions, and thermodynamic parameters (ΔE°, ΔH°, and ΔG°).

To ensure the connectivity between saddle points and corresponding minima, the intrinsic reaction coordinate calculations (IRC)^32,33^ were performed at the M06-2X/6-311+g(2df,2p) level in the mentioned phases. Intending to achieve the accurate and more reliable energetic parameters, i.e. relative energies, we performed higher-level calculations by the CBS-QB3 method for all stationary points. All of the electronic structure calculations in this study were done with the Gaussian 09 program package^34^. The images of all molecular structures were drawn by the Chemcraft program (Version 1.7)^35^.

#### Rate constant calculations

Here, we discuss the main approach and the used theory for the calculation of the rate of multiwell-multichannel reactions. For multi-step reactions, we can write

1 $$A(l)\mathop {\rightleftarrows}\limits^{k_1}_{k_{-1}} B(l)\mathop {\rightleftarrows}\limits^{k_2}_{k_{-2}} C(l)\mathop {\rightleftarrows}\limits^{k_3}_{k_{-3}} D(l)\mathop {\rightleftarrows}\limits^{k_4}_{k_{-4}} E(l),$$

where l stands for liquid, *k*_1_, *k*_2_, *k*_3_, and *k*_4_ are forward rate constants, and *k*_*-*1_, *k*_*-*2_, *k*_-3_, and *k*_-4_ are reverse rate constants. Using the steady-state approximation, the overall rate constant for generation of *B(l)*, *C(l)*, *D(l)*, and *E(l)* are as follows:^36^

2 $$\frac{1}{{k_{obs,B(l)} }} = \frac{1}{{k_{1} }},$$

3 $$\frac{1}{{k_{obs,C(l)} }} = \frac{1}{{k_{1} }} + \frac{{k_{ - 1} }}{{k_{1} k_{2} }},$$

4 $$\frac{1}{{k_{obs,D(l)} }} = \frac{1}{{k_{1} }} + \frac{{k_{ - 1} }}{{k_{1} k_{2} }} + \frac{{k_{ - 1} k_{ - 2} }}{{k_{1} k_{2} k_{3} }},$$

5 $$\frac{1}{{k_{obs,E(l)} }} = \frac{1}{{k_{1} }} + \frac{{k_{ - 1} }}{{k_{1} k_{2} }} + \frac{{k_{ - 1} k_{ - 2} }}{{k_{1} k_{2} k_{3} }} + \frac{{k_{ - 1} k_{ - 2} k_{ - 3} }}{{k_{1} k_{2} k_{3} k_{4} }}.$$

In Eqs. (2)–(5), the forward and reverse rate constants are calculated by employing the Master Equation/Rice–Ramsperger–Kassel–Marcus^37^ (ME/RRKM) theory. In this research the same as gas-phase reactions^38,39^ for calculating the rate constants of multiwell-multichannel reactions, we used the Ssumes program^40^ to solve ME/RRKM for all forward and backward unimolecular reactions. In the framework of RRKM theory, the microcanonical rate constant *k*(*E*) for unimolecular reactions with internal excitation energy *E* is computed by means of very simple expression^41–43^

6 $$k(E) = \frac{{\alpha G^{*} (E - E^{0} )}}{hN(E)}\quad E \ge E^{0} ,$$

where α is the reaction path degeneracy. G*(E − E0) is the integrated density for activated complex without the degree of freedom related to the passage via transition state. *E*^0^ is the critical energy of a reaction. *h* is Planck’s constant. *N(E)* is the density of the rovibrational states of the reactant.

The RRKM calculation input requires the Lenard Jones parameters (collision diameter, σ (Å), and energy parameter, $\varepsilon /{k}_{B}$ (K)) of reactant and third body. The third body is solvent molecules. As far as we know, the Lenard Jones parameters among colliding molecules studying here are not available experimentally but water. The collision diameter, σ (Å), and the energy parameter, $\varepsilon /{k}_{B}$ (K), for water molecule are 2.74 Å and 506 K^44^. The Lennard Jones parameters for 3-hydroxyfuran-2(5H)-one, 3-hydroxyl-1H-pyrrol-2(5H)-one, and 3-hydroxythiophen-2(5H)-one is calculated approximately. First, an approximation method based on the Sanghvi et al.^45^ and Varshni^46^ studies is used for derivation of the critical parameters of the investigating molecules. Finally, we used Tee^47^ relations for extracting the Lennard–Jones parameters of the target molecules using the following equations

7 $$\sigma/\AA = 2.3647 \times \left( {\frac{{T_{c} }}{{P_{c} }}} \right)^{1/3} \, {\text{and}} \, \varepsilon/{\text{k}} = 0.7740 \times T_{c} ,$$

where *T*_*c*_ (in K) and *P*_*c*_ (in atm) are critical temperature and pressure, respectively. The predicted Lennard–Jones parameters for 3-hydroxyfuran-2(5H)-one, 3-hydroxyl-1H-pyrrol-2(5H)-one, and 3-hydroxythiophen-2(5H)-one is 5.34 Å and 519.88 K, 5.44 Å and 580.79 K, and 5.34 Å and 553.67 K, respectively.

## Results and discussion

The intramolecular 1,4-H-shifts in five-member heterocyclic compounds (that are used frequently in drug designing) are modeled with the CBS-QB3, M06-2X/Jun-cc-pVTZ, M06-2X/Jun-cc-pV(T+d)Z, and M06-2X/6-311+g(2df,2p) levels. All total energies, ZPE corrections, thermal energies, enthalpy energies, and Gibbs free energies (in Hartree) are listed in Tables S1-S6 (see Supplementary Information). Also, the computed reverse and overall rate constants (in s^-1^), calculated frequencies (in cm^-1^), and cartesian coordinates are collected in Tables S7-S16 (see Supplementary Information). We simplify the structure drawn in scheme 1by substituting hydrogen atoms in lieu of R_i_ groups. Thus, we use the following compounds.

- 3-Hydroxyl-1H-pyrrol-2(5H)-one.
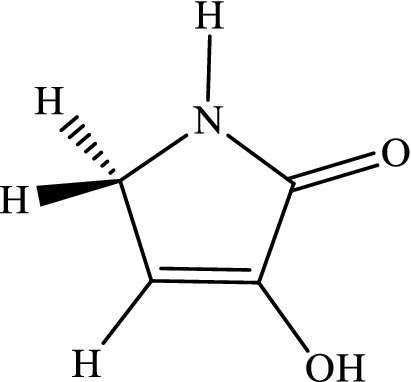
- 3-Hydroxyfuran-2(5H)-one.
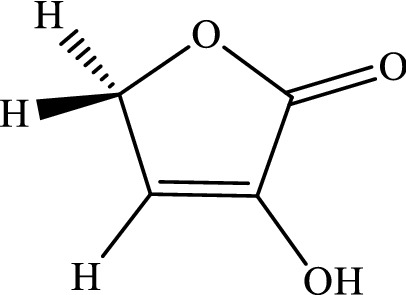
- 3-Hydroxythiophen-2(5H)-one.
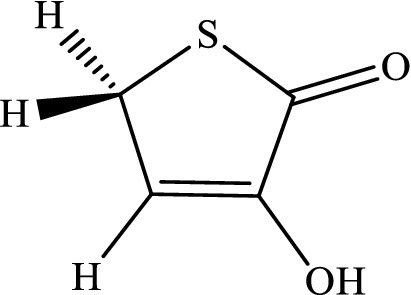

**Scheme 2:**
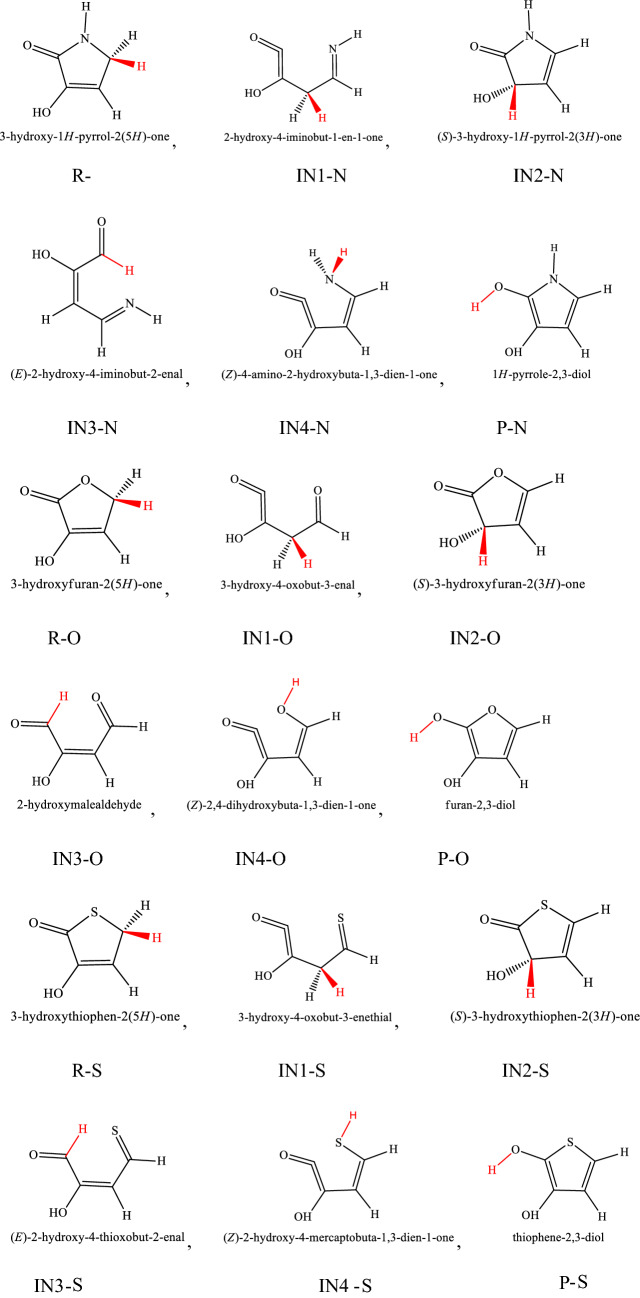
Structures and names of all reactants, intermediates, and products in the suggested pathways of 1,4-hydrogen shifts in 3-hydroxyl-1H-pyrrol-2(5H)-one, 3-hydroxyfuran-2(5H)-one, and 3-hydroxythiophen-2(5H)-one compounds.

The mechanism of 1,4-hydrogen shifts leading to yield the aromatic products is the conventional hydrogen atom transfer. However, all steps of the considered multiwell-multichannel reactions are the conventional hydrogen atom transfer. The most common reaction pathways for the abovementioned compounds are summarized as follows:

$$\begin{array}{*{20}l} {(a){\mkern 1mu} R - X\mathop {\rightleftarrows}\limits^{k_1}_{k_{-1}} IN1 - X\mathop {\rightleftarrows}\limits^{k_2}_{k_{-2}} IN2 - X\mathop {\rightleftarrows}\limits^{k_3}_{k_{-3}} IN3 - X\mathop {\rightleftarrows}\limits^{k_2}_{k_{-2}} P - X} \hfill \\ {(b){\mkern 1mu} R - X\mathop {\rightleftarrows}\limits^{k_5}_{k_-{5}} IN4 - X\mathop {\rightleftarrows}\limits^{k_6}_{k_{-6}} IN3 - X\mathop {\rightleftarrows}\limits^{k_2}_{k_-{2}} P - X} \hfill \\ \end{array} ,$$

where X refers to O, S, and N atoms). In Scheme 2, the molecular structures and names of reactants, intermediates, and products are brought. The prefix Y-N, Y-O, and Y-S denote reactant, intermediates, and product in the reaction pathways of 3-hydroxyl-1H-pyrrol-2(5H)-one, 3-hydroxyfuran-2(5H)-one, and 3-hydroxythiophen-2(5H)-one compounds, respectively.

As far as we are aware, neither experimental nor theoretical rate data are available for the abovementioned 1,4-H shifts. This is the first report for evaluating the mechanism and rate constants of intramolecular 1,4-H shifts of 3-hydroxyl-1H-pyrrol-2(5H)-one, 3-hydroxyfuran-2(5H)-one, and 3-hydroxythiophen-2(5H)-one compounds in the liquid phase. Throughout the text, three consecutive energetics and rate constants belong to the CBS-QB3, M06-2X/Jun-cc-pVTZ (or M06-2X/Jun-cc-pV(T+d)Z for S atom), and M06-2X/6-311+g(2df,2p) levels, respectively.

### Intramolecular H-shifts in 3-hydroxyl-1H-pyrrol-2(5H)-one

**Figure 1:**
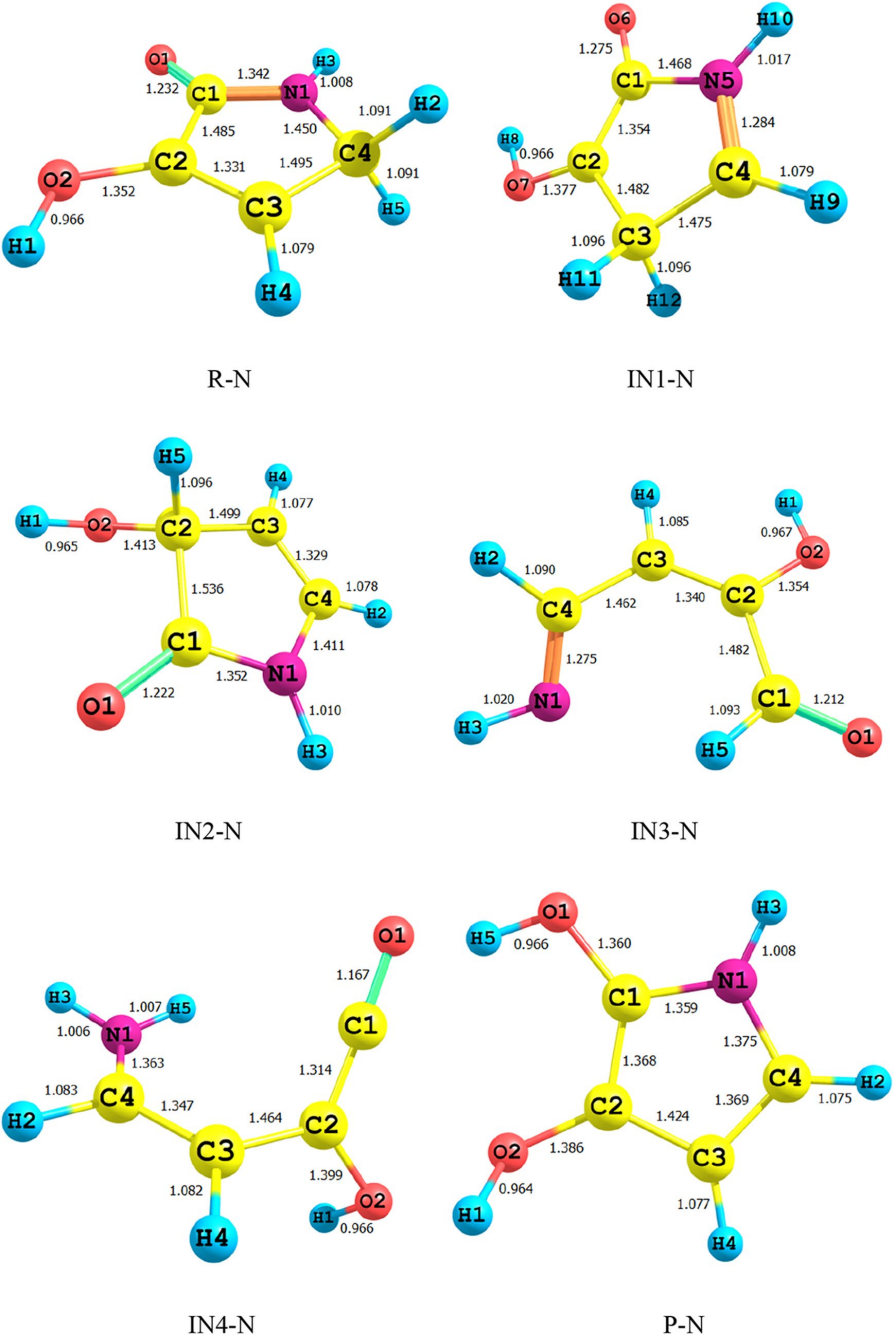

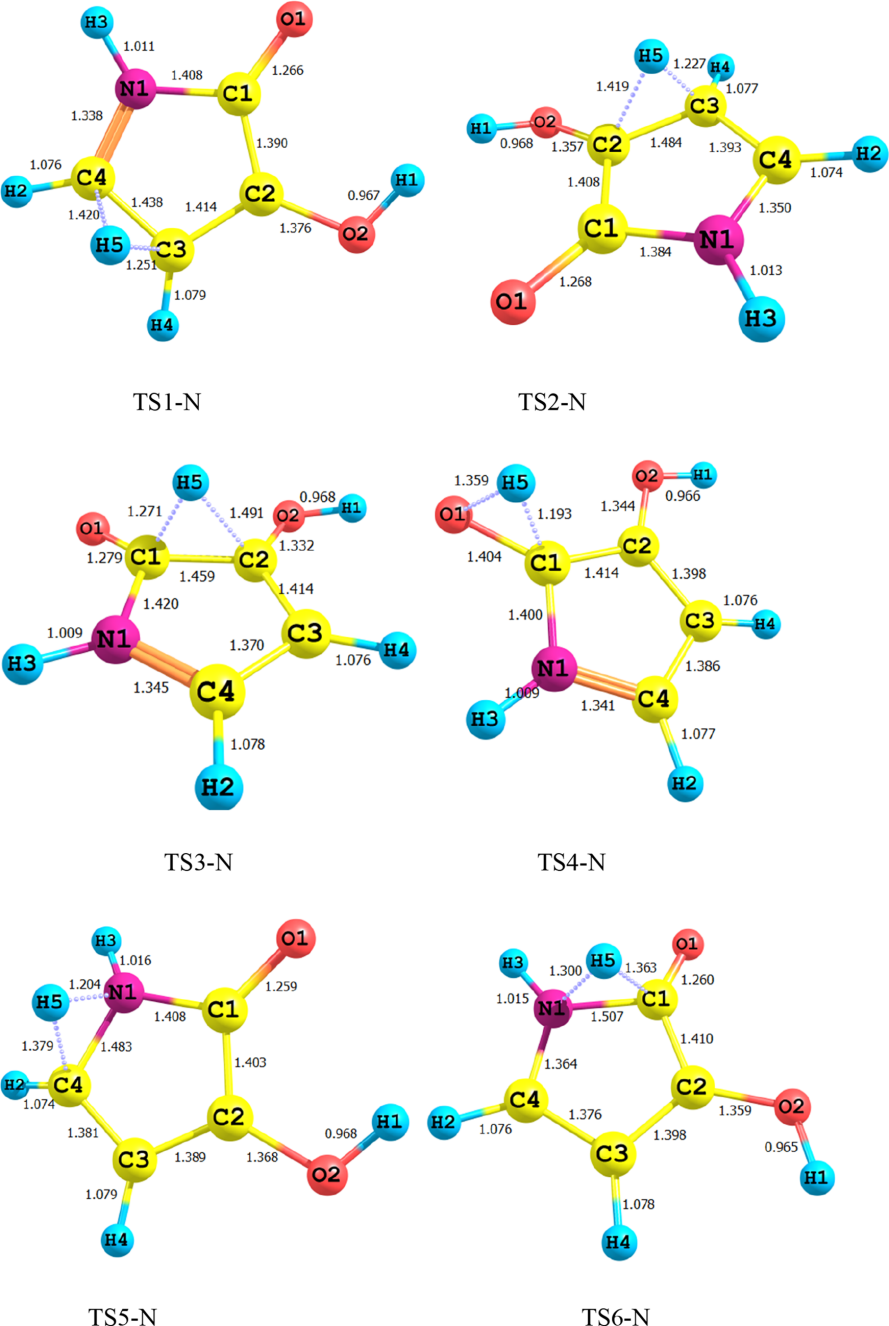
Lowest structures of all stationary points in 3-hydroxyl-1H-pyrrol-2(5H)-one unimolecular reaction obtained at the M06-2X/Jun-cc-pVTZ level.

**Figure 2:**
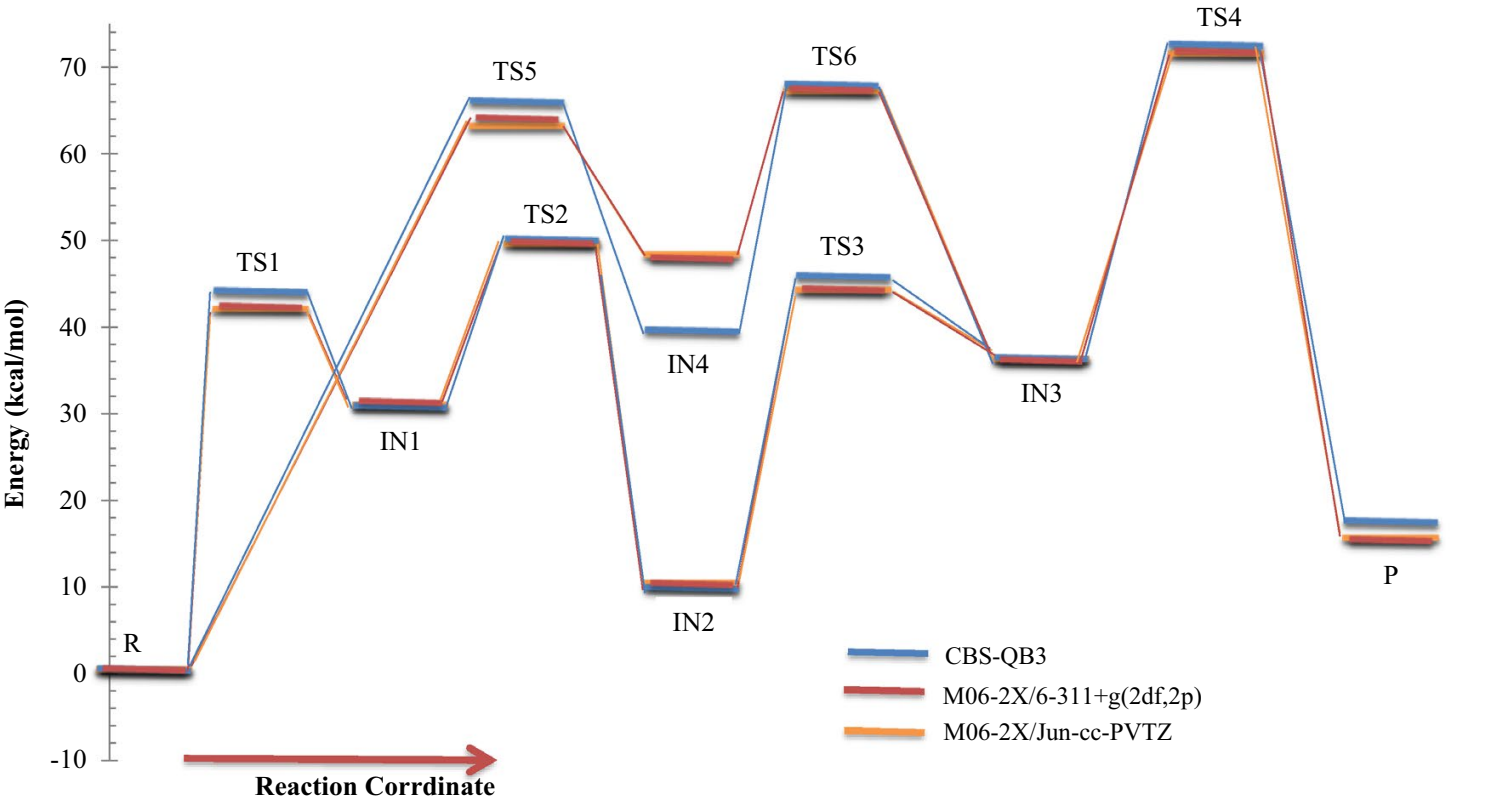
Energy diagram for 3-hydroxyl-1H-pyrrol-2(5H)-one unimolecular reaction computed at the CBS-QB3, M06-2X/Jun-cc-pVTZ, and M06-2X/6-311+g(2df,2p) levels.

**Figure 3:**
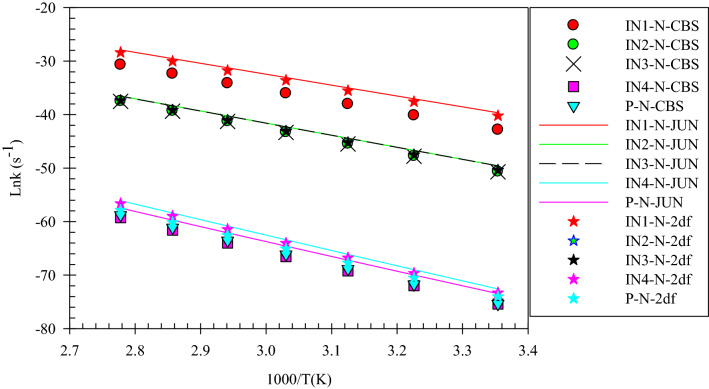
Overall rate constants for production of all minimum stationary points in 3-hydroxyl-1H-pyrrol-2(5H)-one unimolecular reaction calculated at three different levels over 298.15–360 K temperature range.

Figure 1 shows all bond lengths of the stationary point’s structures in 1,4-H shift of 3-hydroxyl-1H-pyrrol-2(5H)-one. The schematic potential energy surface for the multiwell-multichannel unimolecular reaction of 3-hydroxyl-1H-pyrrol-2(5H)-one is sketched in Fig. 2. Graph of overall rate constants for the production of all minimum stationary points is displayed in Fig. 3. Thermodynamic parameters such as thermal energies, enthalpies, and Gibbs free energies, and also relative energies corrected by ZPE for all involved species are listed in Table 1. Tables 2 and 3 contain the computed unimolecular rate constants and half-lifes of the intermediates, respectively. All of the mentioned energetic parameters and rate constants are calculated at the CBS-QB3, M06-2X/Jun-cc-pVTZ, and M06-2X/6-311+g(2df,2p) levels.

**Table 1:** Relative energies corrected by ZPE, thermal energies (ΔE°), enthalpy energies (ΔH°), and Gibbs free energies calculated for all stationary points of 3-hydroxyl-1H-pyrrol-2(5H)-one unimolecular reaction at different levels.

Species	Level	Δ(E+ZPE)	ΔE°	ΔH°	ΔG°
R-N	A	0.00	0.00	0.00	0.00
	B	0.00	0.00	0.00	0.00
	C	0.00	0.00	0.00	0.00
IN1-N	A	30.33	30.46	30.46	30.30
	B	30.50	30.77	30.77	29.39
	C	30.86	30.94	30.94	30.85
IN2-N	A	9.40	9.41	9.41	9.41
	B	9.98	10.04	10.04	9.80
	C	9.84	9.91	9.91	9.71
IN3-N	A	35.91	36.03	36.03	35.59
	B	35.82	36.38	36.38	34.86
	C	35.63	36.25	36.25	34.63
IN4-N	A	39.11	39.27	39.27	39.06
	B	47.87	48.99	48.99	45.98
	C	47.41	48.53	48.53	45.66
TS1-N	A	43.65	43.48	43.48	43.92
	B	41.58	41.43	41.43	41.68
	C	41.83	41.68	41.68	42.01
TS2-N	A	49.60	49.35	49.35	50.00
	B	49.09	48.78	48.78	49.43
	C	49.23	48.96	48.96	49.55
TS3-N	A	45.37	45.03	45.03	45.85
	B	43.79	43.36	43.36	44.24
	C	43.82	43.43	43.43	44.25
TS4-N	A	72.07	71.88	71.88	72.43
	B	71.03	70.85	70.85	71.28
	C	71.25	71.12	71.12	71.47
TS5-N	A	65.54	65.42	65.42	65.78
	B	62.70	62.47	62.47	63.00
	C	63.55	63.52	63.52	63.59
TS6-N	A	67.47	67.32	67.32	67.78
	B	66.69	66.46	66.46	66.97
	C	66.88	66.68	66.68	67.14
P-N	A	17.09	17.32	17.32	17.13
	B	15.18	15.46	15.46	14.99
	C	14.91	15.18	15.18	14.80

A, B, and C are CBS-QB3, M06-2X/Jun-cc-pVTZ, and M06-2X/6-311+g(2df,2p) levels, respectively.

**Table 2:** Unimolecular rate constants (s^−1^) calculated by RRKM theory at the CBS-QB3, M06-2X/Jun-cc-pVTZ, and M06-2X/6-311 + g(2df,2p) levels for all channels of 1,4-H shift of 3-hydroxyl-1H-pyrrol-2(5H)-one.

T/K	CBS-QB3					
	R → 1N1-N	R → 1N4-N	1N1-N → 1N2-N	1N2-N → 1N3-N	1N4-N → 1N3-N	1N3-N → P
298.15	2.49E−19	1.76E−33	1.52E+04	8.50E−02	1.11E+02	1.01E−14
300	3.84E−19	3.06E−33	1.63E+04	9.50E−02	1.27E+02	1.31E−14
310	3.64E−18	5.52E−32	2.32E+04	1.70E−01	2.50E+02	5.16E−14
320	3.02E−17	8.79E−31	3.25E+04	2.97E−01	4.62E+02	1.92E−13
330	2.23E−16	1.24E−29	4.47E+04	5.06E−01	8.08E+02	6.74E−13
340	1.47E−15	1.57E−28	6.05E+04	8.42E−01	1.35E+03	2.25E−12
350	8.73E−15	1.78E−27	8.06E+04	1.37E+00	2.15E+03	7.13E−12
360	4.72E−14	1.82E−26	1.06E+05	2.18E+00	3.30E+03	2.15E−11

T/K	M06-2X/Jun-cc-pVTZ					
	R → 1N1-N	R → 1N4-N	1N1-N → 1N2-N	1N2-N → 1N3-N	1N4-N → 1N3-N	1N3-N → P
298.15	6.03E−18	2.99E−32	2.78E+03	2.89E−02	5.92E+02	1.56E−14
300	9.17E−18	5.33E−32	2.98E+03	3.22E−02	6.31E+02	1.97E−14
310	8.09E−17	1.10E−30	4.23E+03	5.69E−02	8.83E+02	6.85E−14
320	6.27E−16	1.94E−29	5.93E+03	9.81E−02	1.22E+03	2.28E−13
330	4.31E−15	2.91E−28	8.18E+03	1.66E−01	1.66E+03	7.31E−13
340	2.66E−14	3.81E−27	1.11E+04	2.74E−01	2.24E+03	2.25E−12
350	1.48E−13	4.37E−26	1.49E+04	4.44E−01	2.98E+03	6.63E−12
360	7.53E−13	4.45E−25	1.97E+04	7.07E−01	3.92E+03	1.88E−11

T/K	M06-2X/6–311 + g(2df,2p)					
	R → 1N1-N	R → 1N4-N	1N1-N → 1N2-N	1N2-N → 1N3-N	1N4-N → 1N3-N	1N3-N → P
298.15	3.43E−18	1.48E−32	1.58E+04	1.32E−01	4.86E+02	2.35E−14
300	5.23E−18	2.65E−32	1.68E+04	1.46E−01	5.20E+02	2.96E−14
310	4.69E−17	5.60E−31	2.31E+04	2.45E−01	7.45E+02	1.01E−13
320	3.68E−16	1.00E−29	3.14E+04	4.04E−01	1.05E+03	3.37E−13
330	2.57E−15	1.55E−28	4.21E+04	6.54E−01	1.46E+03	1.08E−12
340	1.60E−14	2.08E−27	5.56E+04	1.04E+00	1.99E+03	3.35E−12
350	9.04E−14	2.44E−26	7.26E+04	1.64E+00	2.68E+03	1.00E−11
360	4.65E−13	2.55E−25	9.38E+04	2.53E+00	3.56E+03	2.90E−11

**Table 3:** Half-lifes (in s) of the predicted intermediates in 1,4-H shift of 3-hydroxyl-1H-pyrrol-2(5H)-one at the CBS-QB3, M06-2X/Jun-cc-pVTZ, and M06-2X/6-311 + g(2df,2p) levels.

T/K	CBS-QB3			
	1N1-N	1N2-N	1N3-N	1N4-N
298.15	1.83E−08	7.90E+00	4.18E+01	5.08E−04
300	1.78E−08	7.06E+00	3.67E+01	4.51E−04
310	1.51E−08	3.93E+00	1.84E+01	2.47E−04
320	1.29E−08	2.24E+00	9.56E+00	1.43E−04
330	1.11E−08	1.31E+00	5.12E+00	8.72E−05
340	9.61E−09	7.88E−01	2.82E+00	5.54E−05
350	8.36E−09	4.83E−01	1.60E+00	3.66E−05
360	7.31E−09	3.02E−01	9.26E−01	2.50E−05

T/K	M06-2X/Jun-cc-pVTZ			
	1N1-N	1N2-N	1N3-N	1N4-N
298.15	1.22E−08	2.36E+01	6.28E+01	5.76E−06
300	1.20E−08	2.12E+01	5.55E+01	5.57E−06
310	1.07E−08	1.20E+01	2.91E+01	4.66E−06
320	9.61E−09	6.92E+00	1.57E+01	3.91E−06
330	8.63E−09	4.09E+00	8.69E+00	3.30E−06
340	7.77E−09	2.47E+00	4.94E+00	2.79E−06
350	7.00E−09	1.52E+00	2.88E+00	2.37E−06
360	6.33E−09	9.53E−E−01	1.72E+00	2.02E−06

T/K	M06-2X/6-311 + g(2df,2p)			
	1N1-N	1N2-N	1N3-N	1N4-N
298.15	1.69E−09	5.19E+00	1.67E+01	8.74E−06
300	1.66E−09	4.71E+00	1.48E+01	8.38E−06
310	1.50E−09	2.80E+00	8.11E+00	6.72E−06
320	1.37E−09	1.69E+00	4.54E+00	5.42E−06
330	1.25E−09	1.04E+00	2.60E+00	4.40E−06
340	1.14E−09	6.54E−01	1.52E+00	3.60E−06
350	1.04E−09	4.16E−01	9.09E−01	2.97E−06
360	9.55E−10	2.69E−01	5.53E−01	2.46E−06

According to Fig. 2, the reactant R-N converts to two different intermediates, IN1-N and IN4-N, through two distinguished channels. The calculated relative energies for IN1-N and IN4-N are 30.33, 30.50, and 30.86 kcal mol^−1^ and 39.11, 47.87, and 47.41 kcal mol^−1^ in comparison with the reactant R-N. Our computed rate constants at room temperature for hydrogen transfer from C_α_ to C_β_ and C_α_ to the nitrogen atom (leading to generate IN1-N and IN4-N, respectively) are 2.49E−19, 6.03E−18, 3.43E−18 and s^−1^, and 1.76E−33, 2.99E−32, 1.48E−32 s^−1^, respectively. Comparing the rate constants of IN1-N and IN4-N show that at the initial stages of the reaction concentration of IN4-N is negligible. So, we firstly study path 1. The intermediate, IN1-N can transform into IN2-N via the H atom transfer process by overcoming TS2 with 19.27, 18.59, and 18.37 kcal mol^−1^ energy barrier. This energy barrier is in agreement with the computed rate constant with the value of 1.52E+04, 2.78E+03, and 1.58E+04 s^−1^ at 289.15 K. Also, Fig. 3 illustrates that the production rate of IN2-N is lower than IN1-N as expected for multi-step reactions if the next step barrier is higher than the previous. This statement remains true over the 298.15–360 K temperature range.

As depicted in Fig. 1, IN1-N and IN2-N are two different structural isomers. IN2-N is 20.94, 20.50, and 21.02 kcal mol^−1^ more stable than IN1-N. So, the calculated half-life for IN1-N by a factor of 2.32E−09, 5.17E−10, and 3.26E−10 is lower than IN2-N at room temperature. IN2-N can isomerize into IN3-N through another hydrogen migration with 35.97, 33.81, and 33.98 kcal mol^−1^ energy barrier (TS3). According to the collected rate constants in Table 3, generation rates of IN3-N from IN2-N are very faster than the previous step with the values of 7.90E+00, 2.36E+01, and 5.19E+00 s^−1^ at 289.15 K. On the other hand, Fig. 3 displays that the total generation rates of IN2-N and IN3-N from R-N are equal. This is an expected result because up to the IN3-N formation the rate-determining step is the second step of the reaction.

In the second pathway, the intermediate IN4-N is 38.71, 48.87, and 47.41 kcal mol^−1^ unstable than R-N. This intermediate is produced from R-N by surmounting the high energy barrier of TS5. This path connects to the first pathway through TS6 with an energy barrier of 24.74, 18.82, and 19.74 kcal mol^−1^. IN3-N isomer is more stable than the intermediate IN4-N, due to the formation of the π character bond between nitrogen and C1 atoms and also due to the unsymmetrical distribution of electronic charges in IN4-N.

Finally, IN3-N can convert into the product P-N through the last step of both pathways by overcoming the transition state TS4-N with 34.34, 35.21, and 35.62 kcal mol^−1^ barrier height. Our computed rate for the generation of P-N from IN3-N is 1.01E−14, 1.56E−14, and 2.35E−14 s^−1^ at 289.15 K. It should be noted that this step is the rate-determining step of the overall reaction because the relative energy of TS4-N is 72.07, 71.03, and 71.25 kcal mol^−1^.

It is worth noting that the relative energies of all species, but IN4-N, are not sensitive to the applied methods in water. For IN4-N, the computed energetic parameters have a large difference ~ 8.3–9.7 kcal/mol between the CBS-QB3 and DFT levels. This difference causes to obtain different rates and so half-lifes in the computed methods.

After all, the total rates of all minimum stationary points show that all species produced in the course of reaction have the same behavior from the kinetic point of view (see Fig. 3). The generation rates increase with temperature. Moreover, by emphasizing this point that any change in the drug structure leads to arise a change in its properties. And also, Fig. 3 reveals that 1,4 H-shift of 3-hydroxy-1H-pyrrol-2(5H)-one compound occurs hardly. So, it can be concluded that the drugs based on the considered compound are stable against 1,4-H shifts over the considered temperature range.

### Intramolecular H-shifts in 3-hydroxyfuran-2(5H)-one

**Figure 4:**
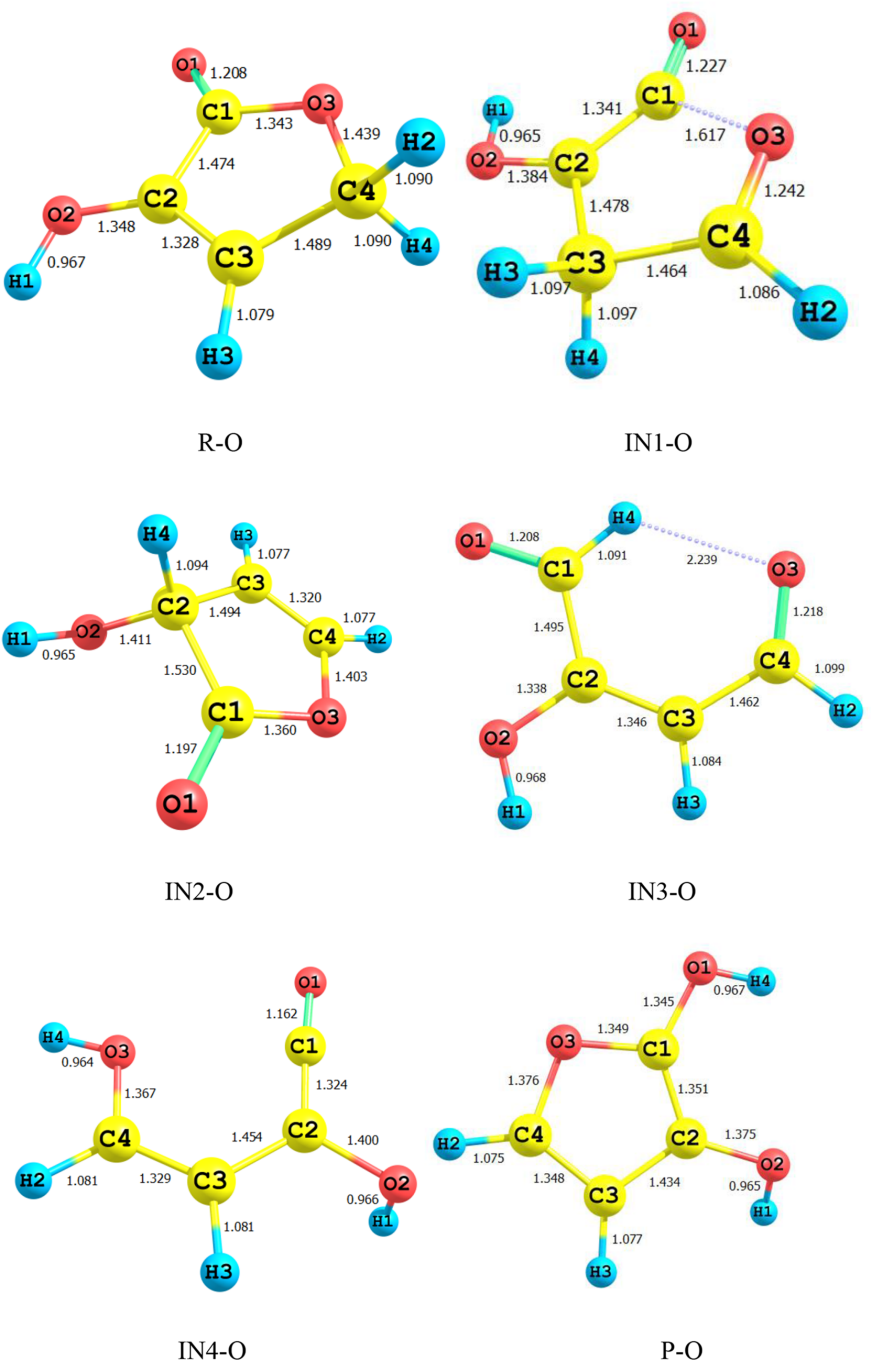

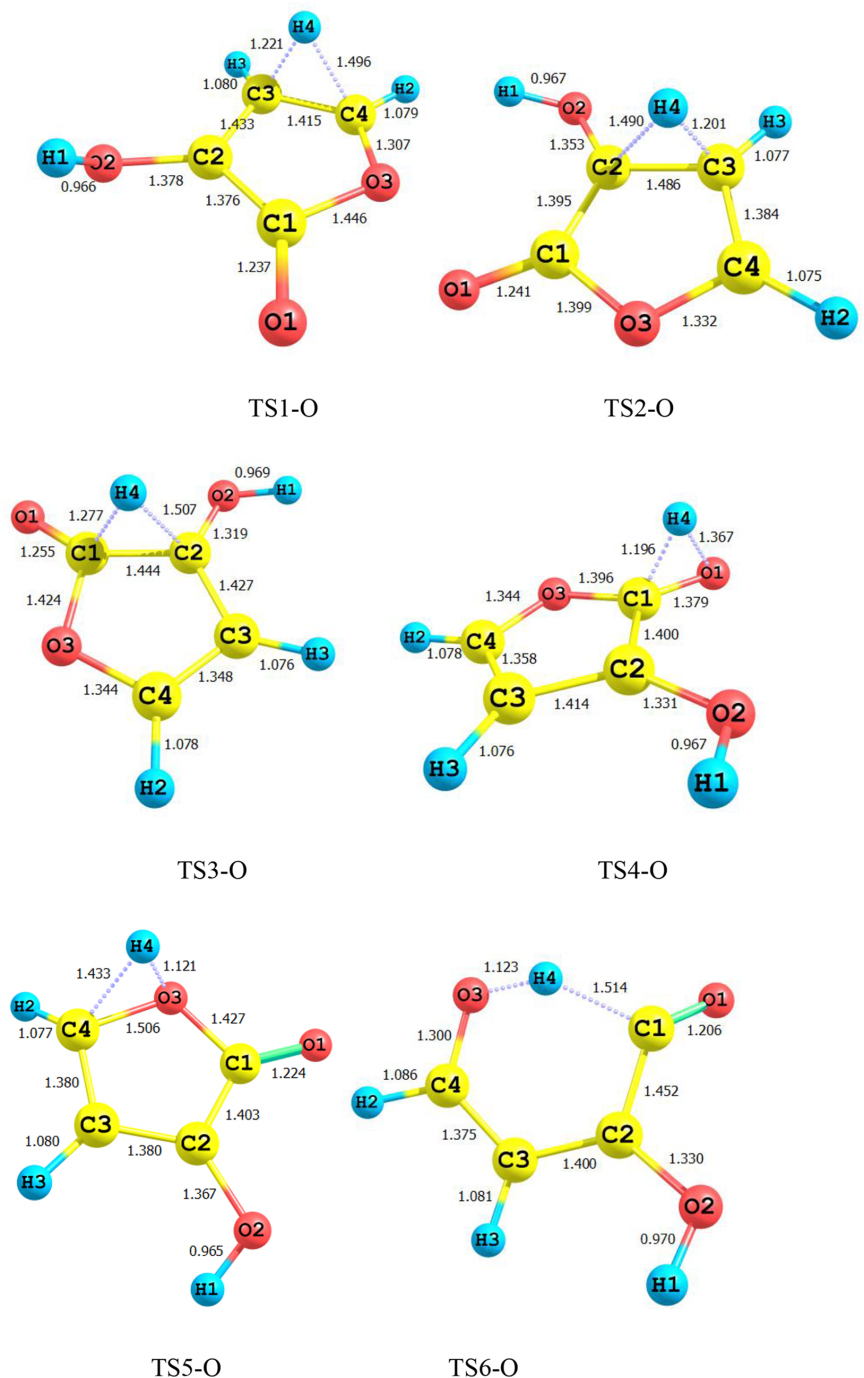
Lowest structures of all stationary points in 3-hydroxyfuran-2(5H)-one unimolecular reaction obtained at the M06-2X/Jun-cc-pVTZ level.

**Figure 5:**
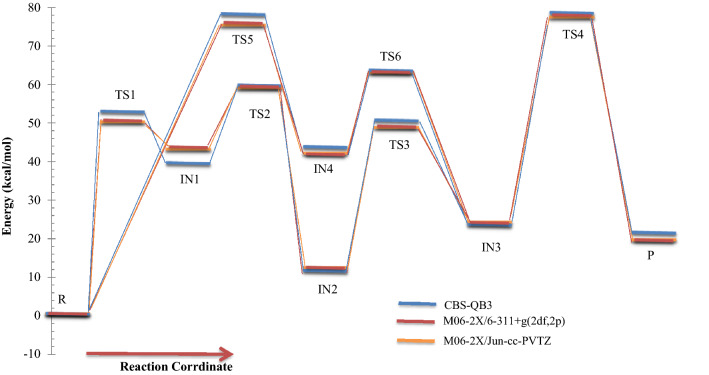
Energy diagram for 3-hydroxyfuran-2(5H)-one unimolecular reaction computed at the CBS-QB3, M06-2X/Jun-cc-pVTZ, and M06-2X/6-311+g(2df,2p) levels.

**Figure 6:**
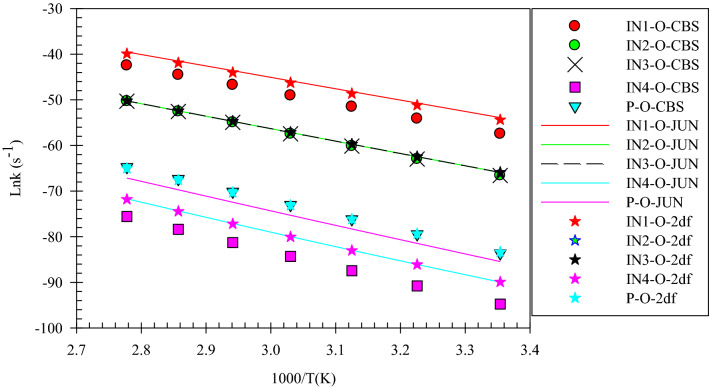
Overall rate constants for production of all minimum stationary points in 3-hydroxyfuran-2(5H)-one unimolecular reaction computed at three different levels over 298.15–360 K temperature range.

Bond lengths of all structures are displayed in Fig. 4. Figure 5 shows the schematic potential energy surface. The calculated overall rate constants are shown in Fig. 6. Tables 4 contain relative energies (corrected by ZPEs), thermodynamic parameters such as thermal energies, enthalpies, Gibbs free energies. Tables 5 and 6 include the computed forward and reverse rate constants and half-lifes of all intermediates.

**Table 4:** Relative energies corrected by ZPE, thermal energies (ΔE°), enthalpy energies (ΔHº), and Gibbs free energies calculated for all stationary points of 3-hydroxyfuran-2(5H)-one unimolecular reaction at different levels.

Species	Level	Δ(E+ZPE)	ΔE°	ΔH°	ΔG°
R-O	A	0.00	0.00	0.00	0.00
	B	0.00	0.00	0.00	0.00
	C	0.00	0.00	0.00	0.00
IN1-O	A	45.34	40.25	40.25	37.53
	B	42.67	42.92	42.92	42.39
	C	43.20	43.90	43.90	42.44
IN2-O	A	11.77	11.20	11.20	10.95
	B	11.98	12.03	12.03	11.80
	C	11.93	12.01	12.01	11.73
IN3-O	A	25.61	23.84	23.84	21.72
	B	23.79	24.16	24.16	23.07
	C	23.58	23.96	23.96	22.86
IN4-O	A	43.68	44.43	44.43	41.52
	B	41.64	42.73	42.73	40.06
	C	41.35	42.42	42.42	39.76
TS1-O	A	53.14	52.58	52.58	52.24
	B	49.76	49.77	49.77	49.73
	C	50.15	50.15	50.15	50.13
TS2-O	A	61.99	59.22	59.22	59.31
	B	58.60	58.56	58.56	58.66
	C	58.90	58.90	58.90	58.92
TS3-O	A	50.95	49.87	49.87	50.48
	B	48.33	47.98	47.98	48.66
	C	48.55	48.22	48.22	48.86
TS4-O	A	80.46	77.97	77.97	78.23
	B	76.94	76.86	76.86	77.06
	C	77.45	77.36	77.36	77.57
TS5-O	A	79.34	77.85	77.85	77.65
	B	74.87	74.99	74.99	74.81
	C	75.43	75.58	75.58	75.34
TS6-O	A	67.51	63.45	63.45	62.55
	B	62.60	62.95	62.95	61.99
	C	62.70	63.08	63.08	62.05
P-O	A	20.00	21.33	21.33	20.79
	B	19.17	19.51	19.51	18.91
	C	19.06	19.39	19.39	18.81

A, B, and C are CBS-QB3, M06-2X/Jun-cc-pVTZ, and M06-2X/6-311+g(2df,2p) levels, respectively.

**Table 5:** Unimolecular rate constants (s^−1^) calculated by RRKM theory at the CBS-QB3, M06-2X/Jun-cc-pVTZ, and M06-2X/6-311+g(2df,2p) levels for all channels of 1,4-H shift of 3-hydroxyfuran-2(5H)-one.

T/K	R → 1N1-O	R → 1N4-O	1N1-O → 1N2-O	1N2-O → 1N3-O	1N4-O → 1N3-O	1N3-O → P
**CBS-QB3**						
298.15	1.09E−25	6.92E−42	2.04E+02	1.03E−02	5.20E+05	1.80E−16
300	1.87E−25	1.26E−41	2.21E+02	1.15E−02	5.69E+05	2.37E−16
310	3.02E−24	3.71E−40	3.33E+02	2.08E−02	8.94E+05	9.81E−16
320	4.14E−23	1.02E−38	4.91E+02	3.67E−02	1.32E+06	3.81E−15
330	4.86E−22	2.37E−37	7.10E+02	6.34E−02	1.85E+06	1.39E−14
340	4.96E−21	4.95E−36	1.01E+03	1.07E−01	2.46E+06	4.80E−14
350	4.46E−20	9.08E−35	1.41E+03	1.78E−01	3.15E+06	1.57E−13
360	3.55E−19	1.48E−33	1.95E+03	2.89E−01	3.91E+06	4.86E−13
**M06-2X/jun-cc-pVTZ**						
298.15	3.85E−24	8.63E−40	5.35E+04	3.99E+00	1.85E+05	5.17E−12
300	6.45E−24	1.61E−39	5.61E+04	4.30E+00	2.05E+05	6.29E−12
310	9.39E−23	4.11E−38	7.23E+04	6.44E+00	3.41E+05	1.80E−11
320	1.16E−21	9.20E−37	9.26E+04	9.57E+00	5.34E+05	5.03E−11
330	1.24E−20	1.82E−35	1.18E+05	1.41E+01	7.88E+05	1.38E−10
340	1.15E−19	3.22E−34	1.50E+05	2.07E+01	1.11E+06	3.68E−10
350	9.39E−19	5.09E−33	1.89E+05	3.01E+01	1.49E+06	9.59E−10
360	6.86E−18	7.21E−32	2.37E+05	4.36E+01	1.92E+06	2.43E−09
**M06-2X/6-311+g(2df,2p)**						
298.15	2.37E−24	8.77E−40	4.38E+04	3.71E+00	1.67E+05	6.95E−14
300	3.98E−24	1.62E−E−39	4.58E+04	3.99E+00	1.85E+05	8.68E−14
310	5.90E−23	4.01E−38	5.82E+04	5.84E+00	3.17E+05	2.85E−13
320	7.40E−22	8.78E−37	7.36E+04	8.49E+00	5.07E+05	9.05E−13
330	7.99E−21	1.71E−35	9.25E+04	1.23E+01	7.64E+05	2.78E−12
340	7.51E−20	2.97E−34	1.16E+05	1.76E+01	1.09E+06	8.29E−12
350	6.23E−19	4.64E−33	1.44E+05	2.50E+01	1.49E+06	2.39E−11
360	4.61E−18	6.51E−32	1.78E+05	3.54E+01	1.95E+06	6.66E−11

**Table 6:** Calculated half-life (in s) for all intermediates in 1,4-H shift of 3-hydroxyfuran-2(5H)-one at the CBS-QB3, M06-2X/Jun-cc-pVTZ, and M06-2X/6-311+g(2df,2p) levels.

T/K	1N1-O	1N2-O	1N3-O	1N4-O
**CBS-QB3**				
298.15	3.70E−07	6.72E+01	2.74E+05	1.33E−06
300	3.59E−07	6.00E+01	2.37E+05	1.22E−06
310	3.04E−07	3.32E+01	1.10E+05	7.75E−07
320	2.58E−07	1.88E+01	5.22E+04	5.25E−07
330	2.21E−07	1.09E+01	2.55E+04	3.75E−07
340	1.90E−07	6.45E+00	1.28E+04	2.82E−07
350	1.64E−07	3.89E+00	6.56E+03	2.20E−07
360	1.42E−07	2.39E+00	3.45E+03	1.77E−07
**M06-2X/Jun-cc-pVTZ**				
298.15	7.83E−11	1.74E−01	1.98E−01	3.75E−06
300	7.78E−11	1.61E−01	1.83E−01	3.39E−06
310	7.55E−11	1.08E−01	1.20E−01	2.03E−06
320	7.31E−11	7.24E−02	8.00E−02	1.30E−06
330	7.08E−11	4.90E−02	5.36E−02	8.80E−07
340	6.86E−11	3.35E−02	3.62E−02	6.27E−07
350	6.64E−11	2.30E−02	2.46E−02	4.67E−07
360	6.42E−11	1.59E−02	1.69E−02	3.60E−07
**M06-2X/6-311+g(2df,2p)**				
298.15	1.54E−10	1.87E−01	1.09E+02	4.16E−06
300	1.54E−10	1.74E−01	9.87E+01	3.74E−06
310	1.49E−10	1.19E−01	5.77E+01	2.19E−06
320	1.45E−10	8.16E−02	3.40E+01	1.37E−06
330	1.41E−10	5.65E−02	2.03E+01	9.08E−07
340	1.37E−10	3.94E−02	1.22E+01	6.35E−07
350	1.33E−10	2.77E−02	7.37E+00	4.66E−07
360	1.30E−10	1.96E−02	4.50E+00	3.55E−07

Isomerization of R-O to IN1-O through TS1-O is slower than the R-N → TS1-N → IN1-N reaction. Because the activation enthalpy of TS1-O is 9.10, 8.34, and 8.47 kcal mol^−1^ higher than TS1-N. The same difference in the barrier heights of mentioned TSs is 9.49, 8.18, and 8.32 kcal mol^−1^. Thus, the IN1-O production rate is 4.38E−07, 6.38E−07, 6.91E−07 times slower than the corresponding rate in 3-hydroxyl-1H-pyrrol-2(5H)-one reaction at room temperature. Also, according to the obtained results IN1-O are 9.79, 12.15, and 12.96 kcal mol^−1^ more unstable than IN-N in standard enthalpy. Also, the computed forward and reverse activation enthalpies for IN1-O formation are 12.33, 6.85, and 6.25 kcal mol^−1^ and 18.97, 15.64, and 15.00 kcal mol^−1^, respectively. These results also are in line with the computed half-life for IN1-O with the value of 3.70E−07, 7.83E−11, and 1.54E−10 s. It is to be noted that the half-life of IN1-O is 2.52E+04, 7.01E+01, and 2.33E+01 times higher than IN1-N at 298.15 K (see Tables 3, 6) because the sum of the revers and forward activation energies for IN1-O → R-O and IN-O → IN2-O channels are higher than that of IN1-N → R-N and IN1-N → IN2-N channels.

Like the previous reaction, the isomerization of reactant R-N to another intermediate that is proceeded by TS5, R-O → TS5-O → IN4-O is very slow due to having high activation enthalpy. The rate of R-O → IN4-O conversion is 6.92E−42, 8.63E−40, and 8.77E−40 s^−1^. The heat of formation of IN4-O is 44.43, 42.73, 42.42 kcal mol^−1^. Also, the difference in heat of formation between IN1-O and IN4-O (ΔH°(IN4-O)–ΔH°(IN1-O)) is 4.18, − 0.19, − 1.48 kcal mol^−1^. The CBS-QB3 method indicates that the equilibrium concentration of IN1-O must be greater than IN4-O. So, it experiences more collisions from ambient molecules. But, the M06-2X method indicates that this statement is true for IN4-O. Also, our computed half-lifes for IN4-O shows that it has a longer lifetime than IN1-O. It seems that if we examine the reaction experimentally, IN4-O may seen due to more half-life, but IN1-O will not observed. Therefore, it can be concluded that the predicted stability by two methods is challenging and for exact judge, it is required to experimental results. On the other hand, the fraction of IN1-O should be low in the course of the reaction, relating to the very fast transformation of this intermediate to IN2-O. The calculated rate constant for IN1-O → IN2-O transformation is 2.04E+02, 5.35E+04, and 4.38E+04 s^−1^. As aforementioned, by increasing reaction steps the rate of reaction decreases. So, the IN2-O formation rate from reactant is slower than IN1-O production (see Fig. 6). The heat of formation of IN2-O is − 16.41, − 18.76, and − 19.94 kcal mol^−1^ lower than the enthalpy of formation of IN1-O. The relatively stable intermediate IN2-O converts to the intermediate IN3-O by TS3. Activation enthalpy for TS3 is 26.03, 23.82, and 24.26 kcal mol^−1^. IN2-O due to having more stability has a higher half-life than IN2-O and IN3.

In the last stage of the reaction IN3-O isomerizes to the final product P-O through hydrogen migration from C1 atom to O1 by TS4-O (see Fig. 5). Activation enthalpy for this process is 54.13, 52.70, and 53.40 kcal mol^−1^. The same as 1,4-H shift of 3-hydroxyl-1H-pyrrol-2(5H)-one this step is the rate-determining step of the overall reaction. The obtained rate constant for the rate-determining step is 1.80E−16, 5.17E−12, and 6.95E−14 s^−1^ at 298.15.

The overall rates of the intermediates and products in the considered 1,4-H shifts (see Figs. 3 and 6) clearly show that 3-hydroxyfuran-2(5H)-one has more chemical resistance (more stable) than 3-hydroxyl-1H-pyrrol-2(5H)-one over the 298.15–360 K temperature range. The substance has a high chemical resistance.

### Intramolecular H-shifts in 3-hydroxythiophen-2(5H)-one

**Figure 7:**
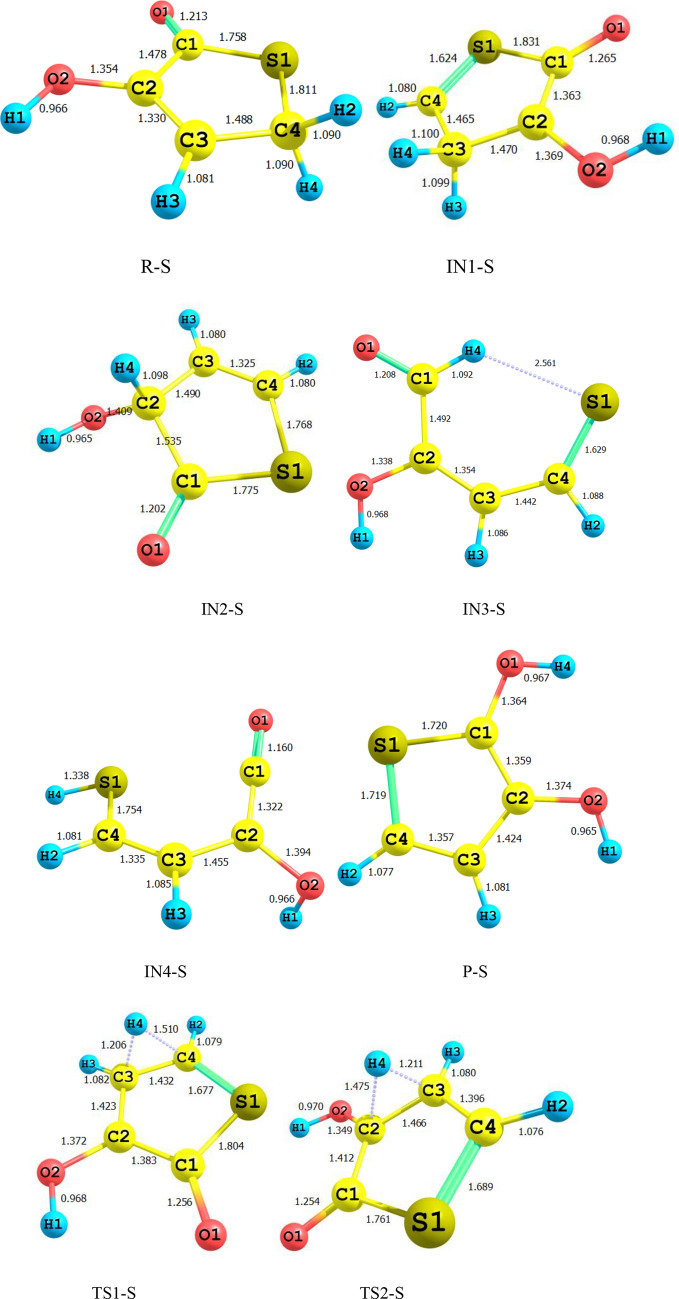

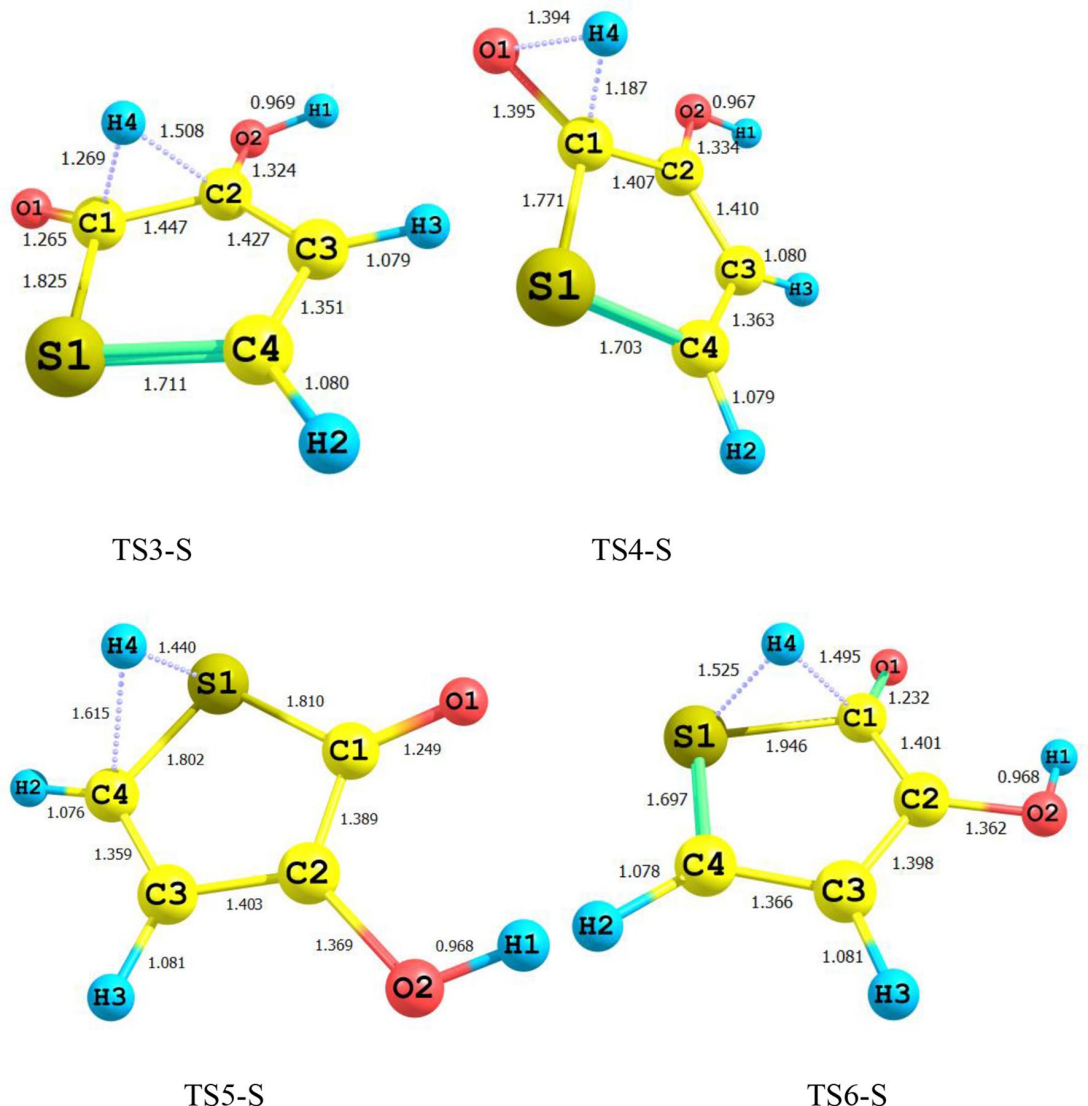
Lowest structures of all stationary points in 3-hydroxythiophen-2(5H)-one unimolecular reaction obtained at the M06-2X/Jun-cc-pV(T+d)Z level.

**Figure 8:**
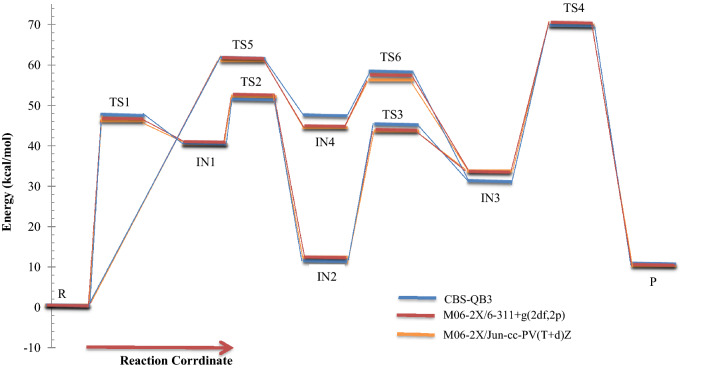
Energy diagram for 3-hydroxythiophen-2(5H)-one unimolecular reaction computed at the CBS-QB3, M06-2X/Jun-cc-pV(T+d)Z, and M06-2X/6-311+g(2df,2p) levels.

**Figure 9:**
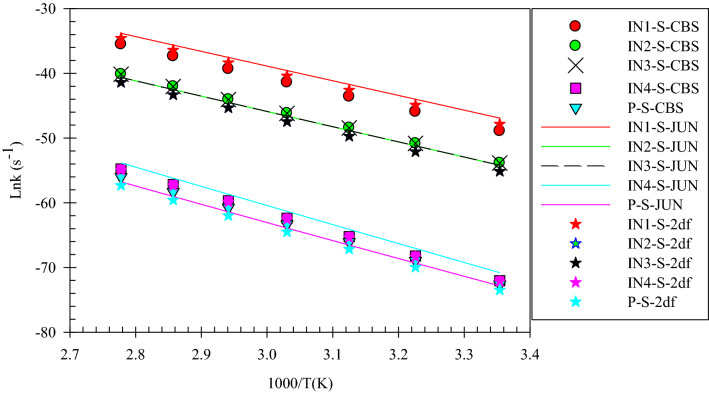
Overall rate constants for production of all minimum stationary points in 3-hydroxythiophen-2(5H)-one unimolecular reaction calculated at three different levels over 298.15–360 K temperature range.

For all structures, the most relevant geometrical parameters are depicted in Fig. 7. Potential energy surface for the 1,4-H shifts in 3- hydroxythiophen-2(5H)-one compound is displayed in Fig. 8. Figure 9 represents the calculated overall rate constants. Table 7 lists the relative energies and thermodynamic parameters such as thermal energies enthalpies, Gibbs free energies for all maximum and minimum points. Unimolecular rate constants for all intermediate and product formation are tabulated in Table 8, and half-lifes of the reactant the intermediates IN1-S, IN2S, and IN3-S are listed in Table 9.

**Table 7:** Relative energies corrected by ZPE, thermal energies (ΔE°), enthalpy energies (ΔHº), and Gibbs free energies calculated for all stationary points of 3-hydroxythiophen-2(5H)-one unimolecular reaction at different levels.

Species	Level	Δ(E+ZPE)	ΔE°	ΔH°	ΔG°
R-S	A	0.00	0.00	0.00	0.00
	B	0.00	0.00	0.00	0.00
	C	0.00	0.00	0.00	0.00
IN1-S	A	40.02	40.29	40.29	39.80
	B	39.91	40.13	40.13	39.78
	C	40.48	40.74	40.74	40.32
IN2-S	A	10.94	11.06	11.06	10.64
	B	11.96	12.10	12.10	11.61
	C	11.93	12.07	12.07	11.58
IN3-S	A	30.75	31.30	31.30	29.22
	B	33.22	33.41	33.41	32.53
	C	33.09	33.29	33.29	32.38
IN4-S	A	47.04	48.09	48.09	45.36
	B	43.96	45.14	45.14	41.67
	C	44.40	45.28	45.28	42.99
TS1-S	A	47.14	47.10	47.10	47.24
	B	45.66	45.56	45.57	45.82
	C	46.24	46.17	46.17	46.38
TS2-S	A	50.95	50.84	50.84	51.16
	B	51.61	51.46	51.46	51.86
	C	52.17	52.03	52.03	52.41
TS3-S	A	44.80	44.53	44.53	45.11
	B	43.14	42.81	42.81	43.51
	C	43.46	43.14	43.14	43.81
TS4-S	A	69.25	69.15	69.15	69.43
	B	69.31	69.19	69.20	69.50
	C	70.01	69.92	69.92	70.18
TS5-S	A	61.19	61.33	61.33	61.18
	B	60.54	60.56	60.56	60.65
	C	61.23	61.27	61.27	61.33
TS6-S	A	57.88	58.06	58.06	57.44
	B	55.79	55.80	55.80	55.87
	C	57.03	57.10	57.10	57.05
P-S	A	10.35	10.73	10.73	10.08
	B	9.78	10.21	10.21	9.37
	C	10.04	10.46	10.46	9.66

A, B, and C are CBS-QB3, M06-2X/Jun-cc-pV(T+d)Z, and M06-2X/6-311+g(2df,2p) levels, respectively.

**Table 8:** Unimolecular rate constants (s^-1^) calculated by RRKM theory at the CBS-QB3, M06-2X/Jun-cc-pV(T + d)Z, and M06-2X/6–311 + g(2df,2p) levels for all channels of 3-hydroxythiophen-2(5H)-one.

T/K	CBS-QB3					
	R → 1N1-S	R → 1N4-S	1N1-S → 1N2-S	1N2-S → 1N3-S	1N4-S → 1N3-S	1N3-S → P
298.15	5.66E−22	5.13E−32	7.30E+07	1.98E+03	1.75E+08	1.43E−09
300	9.13E−22	9.47E−32	7.39E+07	2.06E+03	1.60E+08	1.73E−09
310	1.10E−20	2.31E−30	7.89E+07	2.56E+03	1.45E+08	4.74E−09
320	1.15E−19	4.69E−29	8.41E+07	3.19E+03	1.32E+08	1.26E−08
330	1.04E−18	8.01E−28	8.97E+07	3.96E+03	1.19E+08	3.23E−08
340	8.29E−18	1.17E−26	9.55E+07	4.93E+03	1.08E+08	8.03E−08
350	5.89E−17	1.47E−25	1.02E+08	6.12E+03	9.67E+07	1.94E−07
360	3.77E−16	1.62E−24	1.08E+08	7.61E+03	8.67E+07	4.53E−07

T/K	M06-2X/Jun-cc-pV(T + d)Z					
	R → 1N1-S	R → 1N4-S	1N1-S → 1N2-S	1N2-S → 1N3-S	1N4-S → 1N3-S	1N3-S → P
298.15	4.21E−21	1.77E−31	3.16E+07	4.30E+03	3.43E+06	1.54E−08
300	6.72E−21	3.23E−31	3.22E+07	4.48E+03	3.51E+06	1.81E−08
310	7.68E−20	7.44E−30	3.51E+07	5.59E+03	4.01E+06	4.31E−08
320	7.56E−19	1.43E−28	3.82E+07	6.99E+03	4.54E+06	1.01E−07
330	6.50E−18	2.32E−27	4.17E+07	8.77E+03	5.11E+06	2.34E−07
340	4.93E−17	3.22E−26	4.55E+07	1.10E+04	5.74E+06	5.30E−07
350	3.34E−16	3.89E−25	4.96E+07	1.38E+04	6.41E+06	1.18E−06
360	2.04E−15	4.11E−24	5.41E+07	1.73E+04	7.13E+06	2.56E−06

T/K	M06-2X/6–311 + g(2df,2p)					
	R → 1N1-S	R → 1N4-S	1N1-S → 1N2-S	1N2-S → 1N3-S	1N4-S → 1N3-S	1N3-S → P
298.15	1.65E−21	4.53E−32	2.88E+07	2.98E+03	1.11E+07	1.08E−08
300	2.66E−21	8.34E−32	2.93E+07	3.10E+03	1.15E+07	1.27E−08
310	3.14E−20	2.00E−30	3.19E+07	3.87E+03	1.34E+07	3.03E−08
320	3.18E−19	4.00E−29	3.48E+07	4.85E+03	1.54E+07	7.15E−08
330	2.81E−18	6.74E−28	3.80E+07	6.08E+03	1.77E+07	1.66E−07
340	2.19E−17	9.72E−27	4.14E+07	7.63E+03	2.02E+07	3.77E−07
350	1.52E−16	1.21E−25	4.52E+07	9.59E+03	2.30E+07	8.39E−07
360	9.51E−16	1.32E−24	4.93E+07	1.20E+04	2.60E+07	1.83E−06

**Table 9:** Temperature dependent half-lifes (in s) of the predicted intermediates in 1,4-H shift of 3-hydroxythiophen-2(5H)-one at the CBS-QB3, M06-2X/Jun-cc-pV(T + d)Z, and M06-2X/6–311 + g(2df,2p) levels.

T/K	CBS-QB3			
	1N1-S	1N2-S	1N3-S	1N4-S
298.15	6.78E−11	3.48E−04	1.91E−02	3.86E−09
300	6.76E−11	3.34E−04	1.79E−02	4.23E−09
310	6.67E−11	2.69E−04	1.25E−02	4.67E−09
320	6.59E−11	2.16E−04	8.75E−03	5.14E−09
330	6.52E−11	1.74E−04	6.13E−03	5.71E−09
340	6.45E−11	1.39E−04	4.31E−03	6.30E−09
350	6.39E−11	1.12E−04	3.04E−03	7.04E−09
360	6.34E−11	9.01E−05	2.15E−03	7.86E−09

T/K	M06-2X/Jun-cc-pV(T + d)Z			
	1N1-S	1N2-S	1N3-S	1N4-S
298.15	1.50E−11	1.61E−04	3.24E−04	2.00E−07
300	1.49E−11	1.55E−04	3.07E−04	1.96E−07
310	1.48E−11	1.24E−04	2.32E−04	1.71E−07
320	1.47E−11	9.90E−05	1.76E−04	1.51E−07
330	1.45E−11	7.89E−05	1.33E−04	1.34E−07
340	1.44E−11	6.29E−05	1.01E−04	1.19E−07
350	1.43E−11	5.01E−05	7.73E−05	1.07E−07
360	1.42E−11	3.99E−05	5.92E−05	9.58E−08

T/K	M06-2X/6-311 + g(2df,2p)			
	1N1-S	1N2-S	1N3-S	1N4-S
298.15	1.63E−11	2.33E−04	5.88E−04	6.16E−08
300	1.63E−11	2.23E−04	5.57E−04	5.98E−08
310	1.61E−11	1.79E−04	4.16E−04	5.13E−08
320	1.60E−11	1.43E−04	3.11E−04	4.43E−08
330	1.58E−11	1.14E−04	2.33E−04	3.85E−08
340	1.57E−11	9.07E−05	1.75E−04	3.37E−08
350	1.55E−11	7.22E−05	1.32E−04	2.97E−08
360	1.54E−11	5.74E−05	9.96E−05	2.62E−08

The same as the above-discussed compounds, the most probable pathway is the first route for 1,4-H shifts of 3-hydroxythiophen-2(5H)-one as shown in Fig. 7. In this pathway, the activation free energy of TS1-S is 47.24, 45.82, and 46.38 kcal mol^−1^ to form the intermediate IN1-S. Obtained relative free energy for IN1-S is 39.80, 39.87, and 40.32 kcal mol^−1^. The same values for IN1-N and IN1-O are 30.30, 29.39, and 30.85 and 37.53, 42.39, and 42.44 kcal mol^−1^, respectively. These results indicate that among IN1-N, IN1-O, and IN1-S intermediates the most stable in water is IN1-N. Also IN1-O is more stable than IN1-S. Our computed rate constant for R-S → IN1-S is 5.66E−22, 4.21E−21, and 1.65E−21 s^−1^. Comparing this value with the respective values for R-N → IN1-N and R-O → IN1-O channels reveals that the transformation of R-X to IN1-X in 1,4-H shift of 3-hydroxyfuran-2(5H)-one hardly happens than the others. In the next step, the conversion of IN1-S to the second intermediate, IN2-S, through the hydrogen atom jumping from C2 to C3 via TS2-S requires 11.36, 12.08, and 12.09 kcal mol^−1^ in activation free energy. Our computed unimolecular rate constant for IN1-S → IN2-S channel is 3.58E+05, 5.91E+02, 6.58E+02 times more than the rate of IN1-O → IN2-O channel and is 4.80E+03, 1.14E+04, 1.82E+03 times more than IN1-N transformation to IN2-N at 298.15 K. Newly formed intermediate (IN2-S) is the most stable intermediate among the others by the relative free energy of 10.64, 11.61, and 11.64 kcal mol^−1^. Roughly the same values are observed for IN2-O intermediate, but there is a 1.23, 1.81, and 1.87 kcal mol^−1^ difference between IN2-S and IN2-N that IN2-N is more stable. However, the difference of relative free energy for IN-N and IN1-O is 1.54, 2.00, and 2.2 kcal mol^−1^ that IN1-N is more stable. The intermediate IN2-S transforms into IN3-S with 34.47, 31.9, and 32.23 kcal mol^−1^ free energy barrier (TS3). The predicted rate constant for IN2-S → IN3-S channel is 1.98E+03, 4.30E+03, and 2.98E+03 s^−1^ at 298.15 K.

Finally, consumption of IN3-N intermediate may lead to from the final adduct P through TS4-S with activation free energy by 40.21, 36.97, and 37.80 kcal mol^−1^. Also, the relative energy free energy of TS4-S is 8.8, 7.56, 7.39 kcal mol^−1^ lower than TS4-O and is 3, 1.78, 1.29 kcal mol^−1^ lower than TS4-N. So, This step is faster than the corresponding steps in 3-hydroxyfuran-2 (5H)-one and 3-hydroxyl-1H-pyrrol-2(5H)-one reactions.

About the less important pathway (the second pathway), our results show that the initial step of this path is more suitable in comparison with the 3- hydroxyl-1H-pyrrol-2(5H)-one and 3-hydroxyfuran-2(5H)-one unimolecular reactions. The activation free energy for TS5-S is 4.6, 2.35, and 2.26 kcal mol^−1^ and 16.47, 14.16, 14.01 kcal mol^−1^ lower than that of TS5-N and TS5-O, respectively. Thus, the rate of IN4-S production is 2.91E+01, 5.92E+00, and 3.06E+00 times faster than IN4-N and is 7.41E+09, 2.05E+08, and 5.17E+07 faster than IN4-O at 298.15 K. Also, the second step of this path has the activation free energy of 12.08, 14.20, and 14.06 kcal mol^−1^ that is 16.64, 6.79, and 7.42 kcal mol^−1^ and 8.95, 7.73 and 8.23 kcal mol^−1^ lower than the activation free energy of TS4-N and TS5-N, respectively. Therefore, it will have a faster rate constant for conversion to the third intermediate.

Also, According to Figs. 2, 5 and 8, the intermediate IN2-X (X = N, O, and S) and the product P-X are the most stable compounds after reactant R-X in the 1,4-H shifts of 3-hydroxyl-1H-pyrrol-2(5H)-one, 3-hydroxyfuran-2(5H)-one, and 3-hydroxythiophen-2(5H)-one compounds. It is important to note that the intermediates IN2-X cannot be assumed as a product since it has a small half-life and also the forward and reverse free activation energies of these intermediates are lower than the reverse free activation energies of the products P-X (a reaction with a high saddle point). In fact, the product P-X is the major adduct of all investigated reactions because of their attributed heats of formation and Gibbs free energies of reaction, and having reverse high free energy barriers.

As the last point for expressing more clearly the purpose of this study, we mention some concepts here. Our mean by chemical resistance is that when chemical compounds such as drugs do not react from a specified center in defined conditions, we can say that the center has chemical resistance under specified conditions. Also, it is important to note that reactivity and stability are two contrary challenges. Stable compounds are less reactive. But, the condition in which a compound can be assumed stable must be considered. So, for examining the reactivity of a center, we must simulate the most probable reactions of the center by applying reliable conditions. In the sequel, after constituting the potential energy surface, computing the rate of studying reactions should preferably be done to have complete pieces of information. After that, it can be easily judged whether the reaction occurrs or not (in the designed condition).

In summary of the three last sections, it is preferable to say that the calculated PESs and overall rate constants have useful information for the final judge of the drugs’ stability. As seen in Figs. 3, 6 and 9, conversion of the reactant to the final product encounters with small rate constant that is in line with the high the energy barriers. This creates a chemical resistance against to drugs’ intramolecular changes. Also, the computed forward rate constants uncloak this fact that up to the IN3-X formation may occur at high temperatures, but the computed half-lifes and reverse rate constants indicate that the reaction back to the reactant with high percentage and a very small amount of IN3-X may transform to P-X, which is negligible. Because all of the computed rate constants are small (see Tables 2, 5, 8), in a condition of the body (37 °C), any of these reactions do not occur. Therefore, all investigated target drugs are stable versus the above-discussed reactions in the human body. But, it should be noted that instead of different functional groups which are available in real drugs we substituted hydrogen atoms for simplification. Also, it has been widely proven that the attachment of substituents to chemical compounds facilitates their reactions. And as aforementioned, this study is an example of drug stability evaluation against its possible unimolecular reactions. In general, we can say that if 3-hydroxyfuran-2(5H)-one, 3-hydroxythiophen-2(5H)-one, 3-hydroxy-1H-pyrrol-2 (5H)-one based drugs have the same functional groups, the first is the most stable and the second is more stable than the third.

## Conclusion

A combination of quantum chemical calculations accompanied by the RRKM/Eckart rate constants was used to predict the stability of some drugs. In this respect, the stability of drugs based on a change in their structures was considered mechanistically and kinetically. In the mechanistic part, multiwell-multichannel reactions based on 1,4-H shifts in water were designed for pyrrolidinone-based drugs as an example. So, three target drugs simplified as 3-hydroxy-1H-pyrrol-2 (5H)-one, 3-hydroxyfuran-2(5H)-one, and 3-hydroxythiophen-2(5H)-one were selected. In the other words, 1,4-H shifts in simplified compounds with different heteroatoms were selected for quantum chemical calculations. The PESs for the mentioned compounds were computed by the CBS-QB3 composite method, and the meta hybrid M06-2X method in conjunction with two well-behaved basis sets, Jun-cc-pVTZ (or Jun-cc-pV(T+d)Z) and 6-311+g(2df,2p), in water. Also, for more clarification of the mechanism, we reported the thermodynamic parameters of the mentioned compounds and the associated stationary points in the ground-state potential energy surface using high-level theoretical calculations. In the kinetic part, for the formation and consumption of the intermediates INT1-X–IN4-X, and the product P-X, the steady-state approximation assumption accompanied by the computed thermal rate constants were used to calculate the overall rate constants for all intermediates and the main product over 298.15–360 K temperature range. Therefore, the RRKM theory along with Eckart tunneling correction was utilized to calculate forward and reverse rate constants. Also, the overall rate constants were calculated by using Eqs. (2)–(5). Our calculated potential energy surface accompanied by the predicted rate constants indicated that the main reaction pathway for the formation of P-X was the first pathway and the rate of the second pathway due to having a large energy barrier has a very small contribution to the generation of the product P-X. In addition, the large energy barrier in the reverse direction of the last step, P-X → IN3-X, indicates that the reaction has a small likelihood to return if P-X is formed (see Tables 1, 4, 7).

It is merit pointing out that our results demonstrated that the hetero atom plays a key role in a hydrogen atom jumping on five-membered heterocyclic compounds due to make different energetic results and rate constants in the same carbon skeleton. Therefore, it affects directly the stability of drugs. For example, our results showed that 3-hydroxyfuran-2(5H)-one based drugs are the most stable, and 3-hydroxythiophen-2(5H)-one based drugs are more stable than 3-hydroxy-1H-pyrrol-2 (5H)-one based drugs in water.

## Supplementary Information

Supplementary Tables.
