# Supplementary material for: A computer-aided method for controlling chemical resistance of drugs using RRKM theory in the liquid phase
Source: Sci Rep. 2021 Nov 26;11:22971. doi: 10.1038/s41598-021-01751-z (PMC8626518; doi:10.1038/s41598-021-01751-z)
Supplement: Supplementary file 1 — Supplementary Tables. [file 41598_2021_1751_MOESM1_ESM.docx]

**A computer-aided method for controlling chemical resistance of drugs using RRKM theory in the liquid phase**

Hamed Douroudgari*, Morteza Vahedpour*

Department of Chemistry, University of Zanjan, PO Box 38791-45371, Zanjan, Iran

E-mail:  [douroudgari@znu.ac.ir](mailto:%20douroudgari@znu.ac.ir)

E-mail:  [vahed@znu.ac.ir](mailto:%20vahed@znu.ac.ir)

**Table S1.** Total energies and ZPE corrections (in Hartree ) calculated for all stationary points of 3- hydroxyl-1H-pyrrol-2(5H)-one unimolecular reaction at different levels.

**Table S2.** Total energies and ZPE corrections (in Hartree ) calculated for all stationary points of 3-hydroxyfuran-2(5H)-one unimolecular reaction at different levels.

**Table S3.** Total energies and ZPE corrections (in Hartree ) calculated for all stationary points of 3- hydroxythiophen-2(5H)-one unimolecular reaction at different levels.

**Table S4.** Thermal energies, enthalpy energies, and Gibbs free energies (in Hartree) calculated for all stationary points of 3-hydroxyl-1H-pyrrol-2(5H)-one unimolecular reaction at different levels.

**Table S5.** Thermal energies, enthalpy energies, and Gibbs free energies (in Hartree) calculated for all stationary points of 3-hydroxyfuran-2(5H)-one unimolecular reaction at different levels.

**Table S6.** Thermal energies, enthalpy energies, and Gibbs free energies (in Hartree) calculated for all stationary points of 3- hydroxythiophen-2(5H)-one unimolecular reaction at different levels.

**Table S7**. Reverse rate constants (s^-1^) calculated by RRKM theory at the CBS-QB3, M06-2X/Jun-cc-pVTZ, and M06-2X/6-311+g(2df,2p) levels for all channels of 3-hydroxyl-1H-pyrrol-2(5H)-one unimolecular reaction.

**Table S8**. Reverse rate constants (s^-1^) calculated by RRKM theory at the CBS-QB3, M06-2X/Jun-cc-pVTZ, and M06-2X/6-311+g(2df,2p) levels for all channels of 3-hydroxyfuran-2(5H)-one unimolecular reaction.

**Table S9**. Reverse rate constants (s^-1^) calculated by RRKM theory at the CBS-QB3, M06-2X/Jun-cc-pV(T+d)Z, and M06-2X/6-311+g(2df,2p) levels for all channels of 3- hydroxythiophen-2(5H)-one unimolecular reaction.

**Table S10.** Overall rate constants (s^-1^) calculated by RRKM theory at the CBS-QB3, M06-2X/Jun-cc-pVTZ, and M06-2X/6-311+g(2df,2p) levels for all minimum stationary points of 3-hydroxyl-1H-pyrrol-2(5H)-one unimolecular reaction in water.

**Table S11.** Overall rate constants ( s^-1^) calculated by RRKM theory at the CBS-QB3, M06-2X/Jun-cc-pVTZ, and M06-2X/6-311+g(2df,2p) levels for all minimum stationary points of 3-hydroxyfuran-2(5H)-one unimolecular reaction in water.

**Table S12.** Overall rate constants ( s^-1^) calculated by RRKM theory at the CBS-QB3, M06-2X/Jun-cc-pVTZ, and M06-2X/6-311+g(2df,2p) levels for all minimum stationary points of 3- hydroxythiophen-2(5H)-one unimolecular reaction in water.

**Table S13.** Unscaled frequencies for of all stationary points of 3-hydroxyl-1H-pyrrol-2(5H)-one unimolecular reaction computed at the CBS-QB3 method in water.

**Table S14.** Unscaled frequencies for of all stationary points of 3-hydroxyfuran-2(5H)-one unimolecular reaction computed at the CBS-QB3 method in water.

**Table S15.** Unscaled frequencies for of all stationary points of 3- hydroxythiophen-2(5H)-one unimolecular reaction computed at the CBS-QB3 method in water.

**Table S16.** Cartesian coordinates of all components of 3-hydroxyl-1H-pyrrol-2(5H)-one unimolecular, 3-hydroxyfuran-2(5H)-one, and 3-hydroxythiophen-2(5H)-one reactions calculated at the M06-2X/Jun-cc-pVTZ level in water.

**Table S1.** Total energies and ZPE corrections (in Hartree ) calculated for all stationary points of 3- hydroxyl-1H-pyrrol-2(5H)-one unimolecular reaction at different levels.

| **CBS-QB3** | | |  |  |
| --- | --- | --- | --- | --- |
| Species | E(Hartree) | ZPE(Hartree) | |  |
| R-N | -360.0902 | 0.0903 | |  |
| IN1-N | -360.0675 | 0.0877 | |  |
| IN2-N | -360.1009 | 0.0895 | |  |
| IN3-N | -360.0586 | 0.0881 | |  |
| IN4-N | -360.0535 | 0.0892 | |  |
| P-N | -360.0886 | 0.0891 | |  |
| TS1-N | -360.0463 | 0.0852 | |  |
| TS2-N | -360.0368 | 0.0852 | |  |
| TS3-N | -360.0436 | 0.0853 | |  |
| TS4-N | -360.0010 | 0.0853 | |  |
| TS5-N | -360.0114 | 0.0842 | |  |
| TS6-N | -360.0083 | 0.0834 | |  |
| **M06-2X/Jun-cc-pVTZ** | | |  |  |
| Species | E(Hartree) | ZPE(Hartree) | |  |
| R-N | -360.6499 | 0.0917 | |  |
| IN1-N | -360.5994 | 0.0897 | |  |
| IN2-N | -360.6342 | 0.0918 | |  |
| IN3-N | -360.5917 | 0.0905 | |  |
| IN4-N | -360.5706 | 0.0886 | |  |
| P-N | -360.6250 | 0.0910 | |  |
| TS1-N | -360.5791 | 0.0871 | |  |
| TS2-N | -360.5673 | 0.0872 | |  |
| TS3-N | -360.5763 | 0.0878 | |  |
| TS4-N | -360.5320 | 0.0869 | |  |
| TS5-N | -360.5446 | 0.0862 | |  |
| TS6-N | -360.5379 | 0.0859 | |  |
| **M06-2X/6-311+g(2df,2p)** | | |  |  |
| Species | E(Hartree) | ZPE(Hartree) | | |
| R-N | -360.6347 | 0.0920 | | |
| IN1-N | -360.5835 | 0.0899 | | |
| IN2-N | -360.6188 | 0.0918 | | |
| IN3-N | -360.5764 | 0.0905 | | |
| IN4-N | -360.5560 | 0.0888 | | |
| P-N | -360.6100 | 0.0911 | | |
| TS1-N | -360.5633 | 0.0873 | | |
| TS2-N | -360.5516 | 0.0873 | | |
| TS3-N | -360.5608 | 0.0879 | | |
| TS4-N | -360.5161 | 0.0870 | | |
| TS5-N | -360.5273 | 0.0859 | | |
| TS6-N | -360.5222 | 0.0860 | | |

**Table S2.** Total energies and ZPE corrections (in Hartree ) calculated for all stationary points of 3-hydroxyfuran-2(5H)-one unimolecular reaction at different levels.

| **CBS-QB3** | | | | |  |  |
| --- | --- | --- | --- | --- | --- | --- |
| Species | E(Hartree) | | ZPE(Hartree) |  |  |  |
| R-O | -379.9768 | | 0.0777 |  |  |  |
| IN1-O | -379.9146 | | 0.0744 |  |  |  |
| IN2-O | -379.9591 | | 0.0777 |  |  |  |
| IN3-O | -379.9401 | | 0.0752 |  |  |  |
| IN4-O | -379.9079 | | 0.0746 |  |  |  |
| P-O | -379.9433 | | 0.0766 |  |  |  |
| TS1-O | -379.8932 | | 0.0720 |  |  |  |
| TS2-O | -379.8824 | | 0.0723 |  |  |  |
| TS3-O | -379.8969 | | 0.0730 |  |  |  |
| TS4-O | -379.8524 | | 0.0726 |  |  |  |
| TS5-O | -379.8530 | | 0.0707 |  |  |  |
| TS6-O | -379.8763 | | 0.0698 |  |  |  |
| **M06-2X/Jun-cc-pVTZ** | | | | |  |  |
| Species | E(Hartree) | | ZPE(Hartree) |  |  |  |
| R-O | -380.5118 | | 0.0797 |  |  |  |
| IN1-O | -380.4396 | | 0.0754 |  |  |  |
| IN2-O | -380.4931 | | 0.0800 |  |  |  |
| IN3-O | -380.4710 | | 0.0768 |  |  |  |
| IN4-O | -380.4422 | | 0.0764 |  |  |  |
| P-O | -380.4800 | | 0.0783 |  |  |  |
| TS1-O | -380.4272 | | 0.0743 |  |  |  |
| TS2-O | -380.4130 | | 0.0743 |  |  |  |
| TS3-O | -380.4306 | | 0.0755 |  |  |  |
| TS4-O | -380.3836 | | 0.0741 |  |  |  |
| TS5-O | -380.3854 | | 0.0725 |  |  |  |
| TS6-O | -380.4042 | | 0.0718 |  |  |  |
| **M06-2X/6-311+g(2df,2p)** | | | | | |  |
| Species | E(Hartree) | ZPE(Hartree) | | | | |
| R-O | -380.4952 | 0.0798 | | | | |
| IN1-O | -380.4223 | 0.0757 | | | | |
| IN2-O | -380.4764 | 0.0800 | | | | |
| IN3-O | -380.4547 | 0.0769 | | | | |
| IN4-O | -380.4261 | 0.0766 | | | | |
| P-O | -380.4635 | 0.0785 | | | | |
| TS1-O | -380.4101 | 0.0745 | | | | |
| TS2-O | -380.3959 | 0.0743 | | | | |
| TS3-O | -380.4136 | 0.0755 | | | | |
| TS4-O | -380.3663 | 0.0743 | | | | |
| TS5-O | -380.3679 | 0.0726 | | | | |
| TS6-O | -380.3873 | 0.0718 | | | | |

**Table S3.** Total energies and ZPE corrections (in Hartree ) calculated for all stationary points of 3- hydroxythiophen-2(5H)-one unimolecular reaction at different levels.

| **CBS-QB3** | | | |
| --- | --- | --- | --- |
| Species | E(Hartree) | | ZPE(Hartree) |
| R-S | -702.5817 | | 0.0742 |
| IN1-S | -702.5180 | | 0.0714 |
| IN2-S | -702.5643 | | 0.0740 |
| IN3-S | -702.5327 | | 0.0729 |
| IN4-S | -702.5068 | | 0.0695 |
| P-S | -702.5652 | | 0.0733 |
| TS1-S | -702.5066 | | 0.0693 |
| TS2-S | -702.5005 | | 0.0691 |
| TS3-S | -702.5103 | | 0.0696 |
| TS4-S | -702.4714 | | 0.0694 |
| TS5-S | -702.4842 | | 0.0674 |
| TS6-S | -702.4895 | | 0.0674 |
| **M06-2X/Jun-cc-pV(T+d)Z** | | | |
| Species | E(Hartree) | | ZPE(Hartree) |
| R-S | -703.4779 | | 0.0758 |
| IN1-S | -703.4116 | | 0.0732 |
| IN2-S | -703.4590 | | 0.0760 |
| IN3-S | -703.4238 | | 0.0747 |
| IN4-S | -703.4029 | | 0.0709 |
| P-S | -703.4615 | | 0.0751 |
| TS1-S | -703.4005 | | 0.0713 |
| TS2-S | -703.3907 | | 0.0709 |
| TS3-S | -703.4049 | | 0.0716 |
| TS4-S | -703.3625 | | 0.0709 |
| TS5-S | -703.3751 | | 0.0696 |
| TS6-S | -703.3825 | | 0.0693 |
| **M06-2X/6-311+g(2df,2p)** | | | |
| Species | *E*(Hartree) | *ZPE*(Hartree) | |
| R-S | -703.4594 | 0.0760 | |
| IN1-S | -703.3921 | 0.0732 | |
| IN2-S | -703.4406 | 0.0761 | |
| IN3-S | -703.4055 | 0.0748 | |
| IN4-S | -703.3847 | 0.0720 | |
| P-S | -703.4427 | 0.0752 | |
| TS1-S | -703.3811 | 0.0713 | |
| TS2-S | -703.3713 | 0.0710 | |
| TS3-S | -703.3858 | 0.0716 | |
| TS4-S | -703.3428 | 0.0709 | |
| TS5-S | -703.3555 | 0.0696 | |
| TS6-S | -703.3618 | 0.0693 | |

**Table S4.** Thermal energies, enthalpy energies, and Gibbs free energies (in Hartree) calculated for all stationary points of 3-hydroxyl-1H-pyrrol-2(5H)-one unimolecular reaction at different levels.

| **CBS-QB3** | | | | |
| --- | --- | --- | --- | --- |
| Species | *Eº*(Hartree) | *Hº*(Hartree) | *Gº*(Hartree) |  |
| R-N | -360.1096 | -360.1086 | -360.1459 |  |
| IN1-N | -360.0610 | -360.0601 | -360.0976 |  |
| IN2-N | -360.0946 | -360.0936 | -360.1309 |  |
| IN3-N | -360.0521 | -360.0512 | -360.0892 |  |
| IN4-N | -360.0470 | -360.0460 | -360.0837 |  |
| P-N | -360.0820 | -360.0810 | -360.1186 |  |
| TS1-N | -360.0403 | -360.0393 | -360.0759 |  |
| TS2-N | -360.0309 | -360.0300 | -360.0662 |  |
| TS3-N | -360.0378 | -360.0369 | -360.0729 |  |
| TS4-N | -359.9950 | -359.9941 | -360.0305 |  |
| TS5-N | -360.0053 | -360.0044 | -360.0411 |  |
| TS6-N | -360.0023 | -360.0013 | -360.0379 |  |
| **M06-2X/Jun-cc-pVTZ** | | | | |
| Species | *Eº*(Hartree) | *Hº*(Hartree) | *Gº*(Hartree) |  |
| R-N | -360.5521 | -360.5512 | -360.5881 |  |
| IN1-N | -360.5031 | -360.5021 | -360.5413 |  |
| IN2-N | -360.5361 | -360.5352 | -360.5725 |  |
| IN3-N | -360.4941 | -360.4932 | -360.5325 |  |
| IN4-N | -360.4740 | -360.4731 | -360.5148 |  |
| P-N | -360.5275 | -360.5265 | -360.5642 |  |
| TS1-N | -360.4861 | -360.4851 | -360.5217 |  |
| TS2-N | -360.4744 | -360.4734 | -360.5093 |  |
| TS3-N | -360.4830 | -360.4821 | -360.5176 |  |
| TS4-N | -360.4392 | -360.4382 | -360.4745 |  |
| TS5-N | -360.4525 | -360.4516 | -360.4877 |  |
| TS6-N | -360.4462 | -360.4452 | -360.4814 |  |
| **M06-2X/6-311+g(2df,2p)** | | | | |
| Species | *Eº*(Hartree) | *Hº*(Hartree) | *Gº*(Hartree) |  |
| R-N | -360.5366 | -360.5357 | -360.5725 |  |
| IN1-N | -360.4873 | -360.4864 | -360.5233 |  |
| IN2-N | -360.5208 | -360.5199 | -360.5570 |  |
| IN3-N | -360.4789 | -360.4779 | -360.5173 |  |
| IN4-N | -360.4593 | -360.4584 | -360.4997 |  |
| P-N | -360.5124 | -360.5115 | -360.5489 |  |
| TS1-N | -360.4702 | -360.4693 | -360.5056 |  |
| TS2-N | -360.4586 | -360.4577 | -360.4935 |  |
| TS3-N | -360.4674 | -360.4665 | -360.5020 |  |
| TS4-N | -360.4233 | -360.4223 | -360.4586 |  |
| TS5-N | -360.4354 | -360.4345 | -360.4712 |  |
| TS6-N | -360.4304 | -360.4294 | -360.4655 |  |

**Table S5.** Thermal energies, enthalpy energies, and Gibbs free energies (in Hartree) calculated for all stationary points of 3-hydroxyfuran-2(5H)-one unimolecular reaction at different levels.

| **CBS-QB3** | | | |
| --- | --- | --- | --- |
| Species | *Eº*(Hartree) | *Hº*(Hartree) | *Gº*(Hartree) |
| R-O | -379.9710 | -379.9700 | -380.0064 |
| IN1-O | -379.9068 | -379.9059 | -379.9466 |
| IN2-O | -379.9531 | -379.9522 | -379.9890 |
| IN3-O | -379.9330 | -379.9320 | -379.9718 |
| IN4-O | -379.9002 | -379.8992 | -379.9402 |
| P-O | -379.9370 | -379.9360 | -379.9733 |
| TS1-O | -379.8872 | -379.8862 | -379.9232 |
| TS2-O | -379.8766 | -379.8756 | -379.9119 |
| TS3-O | -379.8915 | -379.8905 | -379.9260 |
| TS4-O | -379.8467 | -379.8458 | -379.8818 |
| TS5-O | -379.8469 | -379.8460 | -379.8827 |
| TS6-O | -379.8698 | -379.8689 | -379.9067 |
| **M06-2X/Jun-cc-pVTZ** | | | |
| Species | *Eº*(Hartree) | *Hº*(Hartree) | *Gº*(Hartree) |
| R-O | -380.4264 | -380.4255 | -380.4616 |
| IN1-O | -380.3580 | -380.3571 | -380.3941 |
| IN2-O | -380.4072 | -380.4063 | -380.4428 |
| IN3-O | -380.3879 | -380.3870 | -380.4249 |
| IN4-O | -380.3583 | -380.3574 | -380.3978 |
| P-O | -380.3953 | -380.3944 | -380.4315 |
| TS1-O | -380.3471 | -380.3462 | -380.3824 |
| TS2-O | -380.3331 | -380.3322 | -380.3682 |
| TS3-O | -380.3500 | -380.3490 | -380.3841 |
| TS4-O | -380.3039 | -380.3030 | -380.3388 |
| TS5-O | -380.3069 | -380.3060 | -380.3424 |
| TS6-O | -380.3261 | -380.3252 | -380.3628 |
| **M06-2X/6-311+g(2df,2p)** | | | |
| Species | *Eº*(Hartree) | *Hº*(Hartree) | *Gº*(Hartree) |
| R-O | -380.4097 | -380.4088 | -380.4449 |
| IN1-O | -380.3398 | -380.3388 | -380.3773 |
| IN2-O | -380.3906 | -380.3896 | -380.4262 |
| IN3-O | -380.3715 | -380.3706 | -380.4085 |
| IN4-O | -380.3421 | -380.3412 | -380.3815 |
| P-O | -380.3788 | -380.3779 | -380.4149 |
| TS1-O | -380.3298 | -380.3289 | -380.3650 |
| TS2-O | -380.3159 | -380.3149 | -380.3510 |
| TS3-O | -380.3329 | -380.3319 | -380.3670 |
| TS4-O | -380.2864 | -380.2855 | -380.3213 |
| TS5-O | -380.2893 | -380.2883 | -380.3248 |
| TS6-O | -380.3092 | -380.3082 | -380.3460 |

**Table S6.** Thermal energies, enthalpy energies, and Gibbs free energies (in Hartree) calculated for all stationary points of 3- hydroxythiophen-2(5H)-one unimolecular reaction at different levels.

| **CBS-QB3** | | | |  |
| --- | --- | --- | --- | --- |
| Species | *Eº*(Hartree) | *Hº*(Hartree) | *Gº*(Hartree) | |
| R-S | -702.5753 | -702.5744 | -702.6123 | |
| IN1-S | -702.5111 | -702.5101 | -702.5489 | |
| IN2-S | -702.5577 | -702.5567 | -702.5953 | |
| IN3-S | -702.5254 | -702.5245 | -702.5657 | |
| IN4-S | -702.4987 | -702.4977 | -702.5400 | |
| P-S | -702.5582 | -702.5573 | -702.5962 | |
| TS1-S | -702.5002 | -702.4993 | -702.5370 | |
| TS2-S | -702.4943 | -702.4933 | -702.5308 | |
| TS3-S | -702.5043 | -702.5034 | -702.5404 | |
| TS4-S | -702.4651 | -702.4642 | -702.5017 | |
| TS5-S | -702.4776 | -702.4766 | -702.5148 | |
| TS6-S | -702.4828 | -702.4818 | -702.5208 | |
| **M06-2X/Jun-cc-pV(T+d)Z** | | | |  |
| Species | *Eº*(Hartree) | *Hº*(Hartree) | *Gº*(Hartree) | |
| R-S | -703.3958 | -703.3948 | -703.4325 | |
| IN1-S | -703.3318 | -703.3309 | -703.3691 | |
| IN2-S | -703.3765 | -703.3755 | -703.4140 | |
| IN3-S | -703.3425 | -703.3416 | -703.3807 | |
| IN4-S | -703.3238 | -703.3229 | -703.3661 | |
| P-S | -703.3795 | -703.3786 | -703.4176 | |
| TS1-S | -703.3231 | -703.3222 | -703.3595 | |
| TS2-S | -703.3138 | -703.3128 | -703.3499 | |
| TS3-S | -703.3275 | -703.3266 | -703.3632 | |
| TS4-S | -703.2855 | -703.2845 | -703.3217 | |
| TS5-S | -703.2993 | -703.2983 | -703.3358 | |
| TS6-S | -703.3068 | -703.3059 | -703.3435 | |
| **M06-2X/6-311+g(2df,2p)** | | | |  |
| Species | *Eº*(Hartree) | *Hº*(Hartree) | *Gº*(Hartree) | |
| R-S | -703.3772 | -703.3763 | -703.4139 | |
| IN1-S | -703.3123 | -703.3113 | -703.3497 | |
| IN2-S | -703.3580 | -703.3570 | -703.3954 | |
| IN3-S | -703.3242 | -703.3232 | -703.3623 | |
| IN4-S | -703.3051 | -703.3041 | -703.3454 | |
| P-S | -703.3605 | -703.3596 | -703.3985 | |
| TS1-S | -703.3036 | -703.3027 | -703.3400 | |
| TS2-S | -703.2943 | -703.2934 | -703.3304 | |
| TS3-S | -703.3085 | -703.3075 | -703.3441 | |
| TS4-S | -703.2658 | -703.2649 | -703.3021 | |
| TS5-S | -703.2796 | -703.2786 | -703.3162 | |
| TS6-S | -703.2862 | -703.2853 | -703.3230 | |

**Table S7**. Reverse rate constants (s^-1^) calculated by RRKM theory at the CBS-QB3, M06-2X/Jun-cc-pVTZ, and M06-2X/6-311+g(2df,2p) levels for all channels of 3-hydroxyl-1H-pyrrol-2(5H)-one unimolecular reaction.

| **CBS-QB3** | | | | | |  |
| --- | --- | --- | --- | --- | --- | --- |
| T/K | 1N1-N 🡪R | 1N4-N 🡪R | 1N2-N 🡪1N1-N | 1N3-N 🡪1N2-N | 1N3-N 🡪1N4-N | P 🡪1N3-N |
| 298.15 | 3.78E+07 | 1.25E+03 | 2.77E-03 | 1.66E-02 | 2.94E-12 | 5.28E-24 |
| 300 | 3.90E+07 | 1.41E+03 | 3.15E-03 | 1.89E-02 | 3.75E-12 | 7.62E-24 |
| 310 | 4.59E+07 | 2.55E+03 | 6.11E-03 | 3.76E-02 | 1.34E-11 | 5.22E-23 |
| 320 | 5.36E+07 | 4.38E+03 | 1.15E-02 | 7.25E-02 | 4.52E-11 | 3.24E-22 |
| 330 | 6.23E+07 | 7.14E+03 | 2.10E-02 | 1.35E-01 | 1.43E-10 | 1.84E-21 |
| 340 | 7.20E+07 | 1.12E+04 | 3.74E-02 | 2.46E-01 | 4.27E-10 | 9.64E-21 |
| 350 | 8.28E+07 | 1.68E+04 | 6.50E-02 | 4.34E-01 | 1.21E-09 | 4.68E-20 |
| 360 | 9.47E+07 | 2.44E+04 | 1.10E-01 | 7.48E-01 | 3.29E-09 | 2.12E-19 |
| **M06-2X/Jun-cc-pVTZ** | | | | | |  |
| T/K | 1N1-N 🡪R | 1N4-N 🡪R | 1N2-N 🡪1N1-N | 1N3-N 🡪1N2-N | 1N3-N 🡪1N4-N | P 🡪1N3-N |
| 298.15 | 5.66E+07 | 1.20E+05 | 4.81E-04 | 1.10E-02 | 6.43E-13 | 3.32E-24 |
| 300 | 5.78E+07 | 1.24E+05 | 5.46E-04 | 1.25E-02 | 8.22E-13 | 4.75E-24 |
| 310 | 6.46E+07 | 1.48E+05 | 1.07E-03 | 2.38E-02 | 2.97E-12 | 3.08E-23 |
| 320 | 7.21E+07 | 1.76E+05 | 2.02E-03 | 4.42E-02 | 1.00E-11 | 1.82E-22 |
| 330 | 8.03E+07 | 2.09E+05 | 3.73E-03 | 7.98E-02 | 3.20E-11 | 9.93E-22 |
| 340 | 8.92E+07 | 2.46E+05 | 6.71E-03 | 1.40E-01 | 9.62E-11 | 5.01E-21 |
| 350 | 9.90E+07 | 2.90E+05 | 1.18E-02 | 2.41E-01 | 2.75E-10 | 2.35E-20 |
| 360 | 1.09E+08 | 3.39E+05 | 2.03E-02 | 4.03E-01 | 7.49E-10 | 1.04E-19 |
| **M06-2X/6-311+g(2df,2p)** | | | | | |  |
| T/K | 1N1-N 🡪R | 1N4-N 🡪R | 1N2-N 🡪1N1-N | 1N3-N 🡪1N2-N | 1N3-N 🡪1N4-N | P 🡪1N3-N |
| 298.15 | 4.10E+08 | 7.88E+04 | 1.36E-03 | 4.16E-02 | 8.88E-13 | 4.60E-24 |
| 300 | 4.18E+08 | 8.22E+04 | 1.54E-03 | 4.67E-02 | 1.13E-12 | 6.58E-24 |
| 310 | 4.61E+08 | 1.02E+05 | 2.88E-03 | 8.55E-02 | 4.12E-12 | 4.35E-23 |
| 320 | 5.07E+08 | 1.27E+05 | 5.27E-03 | 1.53E-01 | 1.41E-11 | 2.63E-22 |
| 330 | 5.56E+08 | 1.56E+05 | 9.43E-03 | 2.66E-01 | 4.55E-11 | 1.47E-21 |
| 340 | 6.09E+08 | 1.90E+05 | 1.65E-02 | 4.55E-01 | 1.39E-10 | 7.59E-21 |
| 350 | 6.66E+08 | 2.31E+05 | 2.84E-02 | 7.62E-01 | 4.06E-10 | 3.67E-20 |
| 360 | 7.26E+08 | 2.78E+05 | 4.80E-02 | 1.25E+00 | 1.13E-09 | 1.66E-19 |

**Table S8**. Reverse rate constants (s^-1^) calculated by RRKM theory at the CBS-QB3, M06-2X/Jun-cc-pVTZ, and M06-2X/6-311+g(2df,2p) levels for all channels of 3-hydroxyfuran-2(5H)-one unimolecular reaction.

| **CBS-QB3** | | | | | |  |
| --- | --- | --- | --- | --- | --- | --- |
| T/K | 1N1-O 🡪R | 1N4-O 🡪R | 1N2-O 🡪1N1-O | 1N3-O 🡪1N2-O | 1N3-O 🡪1N4-O | P 🡪1N3-O |
| 298.15 | 1.87E+06 | 4.98E-01 | 1.84E-05 | 2.52E-06 | 1.77E-09 | 1.37E-24 |
| 300 | 1.93E+06 | 5.85E-01 | 2.13E-05 | 2.92E-06 | 2.14E-09 | 1.96E-24 |
| 310 | 2.28E+06 | 1.31E+00 | 4.53E-05 | 6.31E-06 | 5.92E-09 | 1.29E-23 |
| 320 | 2.68E+06 | 2.65E+00 | 9.35E-05 | 1.33E-05 | 1.56E-08 | 7.78E-23 |
| 330 | 3.14E+06 | 4.95E+00 | 1.87E-04 | 2.71E-05 | 3.98E-08 | 4.30E-22 |
| 340 | 3.65E+06 | 8.62E+00 | 3.64E-04 | 5.41E-05 | 9.73E-08 | 2.20E-21 |
| 350 | 4.22E+06 | 1.41E+01 | 6.89E-04 | 1.05E-04 | 2.30E-07 | 1.05E-20 |
| 360 | 4.87E+06 | 2.20E+01 | 1.27E-03 | 2.01E-04 | 5.26E-07 | 4.66E-20 |
| **M06-2X/Jun-cc-pVTZ** | | | | | |  |
| T/K | 1N1-O 🡪R | 1N4-O 🡪R | 1N2-O 🡪1N1-O | 1N3-O 🡪1N2-O | 1N3-O 🡪1N4-O | P 🡪1N3-O |
| 298.15 | 8.86E+09 | 9.57E-01 | 1.78E-03 | 3.50E+00 | 1.11E-04 | 3.10E-21 |
| 300 | 8.91E+09 | 1.12E+00 | 1.99E-03 | 3.79E+00 | 1.28E-04 | 4.24E-21 |
| 310 | 9.19E+09 | 2.53E+00 | 3.60E-03 | 5.75E+00 | 2.67E-04 | 2.22E-20 |
| 320 | 9.48E+09 | 5.20E+00 | 6.43E-03 | 8.66E+00 | 5.46E-04 | 1.08E-19 |
| 330 | 9.79E+09 | 9.86E+00 | 1.13E-02 | 1.29E+01 | 1.09E-03 | 4.97E-19 |
| 340 | 1.01E+10 | 1.74E+01 | 1.96E-02 | 1.91E+01 | 2.13E-03 | 2.14E-18 |
| 350 | 1.04E+10 | 2.90E+01 | 3.36E-02 | 2.81E+01 | 4.07E-03 | 8.75E-18 |
| 360 | 1.08E+10 | 4.60E+01 | 5.67E-02 | 4.10E+01 | 7.66E-03 | 3.39E-17 |
| **M06-2X/6-311+g(2df,2p)** | | | | | |  |
| T/K | 1N1-O 🡪R | 1N4-O 🡪R | 1N2-O 🡪1N1-O | 1N3-O 🡪1N2-O | 1N3-O 🡪1N4-O | P 🡪1N3-O |
| 298.15 | 4.49E+09 | 7.23E-01 | 1.50E-03 | 6.35E-03 | 8.71E-07 | 1.39E-22 |
| 300 | 4.51E+09 | 8.54E-01 | 1.67E-03 | 7.02E-03 | 1.02E-06 | 1.93E-22 |
| 310 | 4.64E+09 | 1.99E+00 | 2.96E-03 | 1.20E-02 | 2.35E-06 | 1.10E-21 |
| 320 | 4.77E+09 | 4.21E+00 | 5.16E-03 | 2.04E-02 | 5.29E-06 | 5.79E-21 |
| 330 | 4.91E+09 | 8.20E+00 | 8.87E-03 | 3.42E-02 | 1.16E-05 | 2.86E-20 |
| 340 | 5.05E+09 | 1.48E+01 | 1.50E-02 | 5.69E-02 | 2.50E-05 | 1.32E-19 |
| 350 | 5.20E+09 | 2.52E+01 | 2.52E-02 | 9.40E-02 | 5.26E-05 | 5.78E-19 |
| 360 | 5.35E+09 | 4.05E+01 | 4.16E-02 | 1.54E-01 | 1.08E-04 | 2.39E-18 |

**Table S9**. Reverse rate constants (s^-1^) calculated by RRKM theory at the CBS-QB3, M06-2X/Jun-cc-pV(T+d)Z, and M06-2X/6-311+g(2df,2p) levels for all channels of 3- hydroxythiophen-2(5H)-one unimolecular reaction.

| **CBS-QB3** | | | | | |  |
| --- | --- | --- | --- | --- | --- | --- |
| T/K | 1N1-S 🡪R | 1N4-S 🡪R | 1N2-S 🡪1N1-S | 1N3-S 🡪1N2-S | 1N3-S 🡪1N4-S | P 🡪1N3-S |
| 298.15 | 1.01E+10 | 7.85E+05 | 1.10E+01 | 3.63E+01 | 9.71E-04 | 1.23E-18 |
| 300 | 1.02E+10 | 8.16E+05 | 1.18E+01 | 3.87E+01 | 1.11E-03 | 1.64E-18 |
| 310 | 1.03E+10 | 9.99E+05 | 1.65E+01 | 5.54E+01 | 2.22E-03 | 7.38E-18 |
| 320 | 1.04E+10 | 1.21E+06 | 2.31E+01 | 7.92E+01 | 4.36E-03 | 3.11E-17 |
| 330 | 1.05E+10 | 1.46E+06 | 3.23E+01 | 1.13E+02 | 8.40E-03 | 1.24E-16 |
| 340 | 1.07E+10 | 1.75E+06 | 4.48E+01 | 1.61E+02 | 1.58E-02 | 4.68E-16 |
| 350 | 1.07E+10 | 2.08E+06 | 6.21E+01 | 2.28E+02 | 2.93E-02 | 1.68E-15 |
| 360 | 1.08E+10 | 2.46E+06 | 8.56E+01 | 3.22E+02 | 5.33E-02 | 5.72E-15 |
| **M06-2X/Jun-cc-pVTZ** | | | | | |  |
| T/K | 1N1-S 🡪R | 1N4-S 🡪R | 1N2-S 🡪1N1-S | 1N3-S 🡪1N2-S | 1N3-S 🡪1N4-S | P 🡪1N3-S |
| 298.15 | 4.63E+10 | 2.90E+04 | 3.80E+00 | 2.14E+03 | 3.35E-02 | 3.18E-18 |
| 300 | 4.64E+10 | 3.04E+04 | 4.09E+00 | 2.26E+03 | 3.73E-02 | 4.18E-18 |
| 310 | 4.68E+10 | 3.86E+04 | 6.01E+00 | 2.98E+03 | 6.61E-02 | 1.77E-17 |
| 320 | 4.73E+10 | 4.84E+04 | 8.81E+00 | 3.94E+03 | 1.15E-01 | 7.12E-17 |
| 330 | 4.77E+10 | 6.01E+04 | 1.29E+01 | 5.20E+03 | 1.98E-01 | 2.71E-16 |
| 340 | 4.81E+10 | 7.39E+04 | 1.87E+01 | 6.83E+03 | 3.35E-01 | 9.81E-16 |
| 350 | 4.84E+10 | 9.00E+04 | 2.70E+01 | 8.96E+03 | 5.59E-01 | 3.39E-15 |
| 360 | 4.88E+10 | 1.09E+05 | 3.88E+01 | 1.17E+04 | 9.18E-01 | 1.12E-14 |
| **M06-2X/6-311+g(2df,2p)** | | | | | |  |
| T/K | 1N1-S 🡪R | 1N4-S 🡪R | 1N2-S 🡪1N1-S | 1N3-S 🡪1N2-S | 1N3-S 🡪1N4-S | P 🡪1N3-S |
| 298.15 | 4.24E+10 | 1.16E+05 | 2.54E+00 | 1.18E+03 | 1.21E-02 | 2.32E-18 |
| 300 | 4.25E+10 | 1.22E+05 | 2.72E+00 | 1.25E+03 | 1.36E-02 | 3.05E-18 |
| 310 | 4.30E+10 | 1.57E+05 | 4.00E+00 | 1.67E+03 | 2.49E-02 | 1.30E-17 |
| 320 | 4.34E+10 | 1.99E+05 | 5.86E+00 | 2.23E+03 | 4.46E-02 | 5.23E-17 |
| 330 | 4.38E+10 | 2.50E+05 | 8.55E+00 | 2.98E+03 | 7.88E-02 | 2.00E-16 |
| 340 | 4.42E+10 | 3.10E+05 | 1.24E+01 | 3.96E+03 | 1.37E-01 | 7.24E-16 |
| 350 | 4.46E+10 | 3.82E+05 | 1.79E+01 | 5.26E+03 | 2.34E-01 | 2.51E-15 |
| 360 | 4.49E+10 | 4.66E+05 | 2.58E+01 | 6.96E+03 | 3.93E-01 | 8.32E-15 |

**Table S10.** Overall rate constants (s^-1^) calculated by RRKM theory at the CBS-QB3, M06-2X/Jun-cc-pVTZ, and M06-2X/6-311+g(2df,2p) levels for all minimum stationary points of 3-hydroxyl-1H-pyrrol-2(5H)-one unimolecular reaction in water.

| **CBS-QB3** | | | | | |
| --- | --- | --- | --- | --- | --- |
| T/K | R🡪IN1-N | R🡪IN2-N | R🡪IN3-N | R🡪IN4-N | R🡪P-N |
| 298.15 | 2.49E-19 | 9.99E-23 | 9.68E-23 | 1.76E-33 | 1.87E-33 |
| 300 | 3.84E-19 | 1.60E-22 | 1.55E-22 | 3.06E-33 | 3.35E-33 |
| 310 | 3.64E-18 | 1.84E-21 | 1.78E-21 | 5.52E-32 | 7.02E-32 |
| 320 | 3.02E-17 | 1.83E-20 | 1.76E-20 | 8.79E-31 | 1.25E-30 |
| 330 | 2.23E-16 | 1.60E-19 | 1.53E-19 | 1.24E-29 | 1.91E-29 |
| 340 | 1.47E-15 | 1.23E-18 | 1.18E-18 | 1.57E-28 | 2.54E-28 |
| 350 | 8.73E-15 | 8.49E-18 | 8.10E-18 | 1.78E-27 | 2.94E-27 |
| 360 | 4.72E-14 | 5.27E-17 | 5.02E-17 | 1.82E-26 | 3.01E-26 |
| **M06-2X/Jun-cc-pVTZ** | | | | | |
| T/K | R🡪IN1-N | R🡪IN2-N | R🡪IN3-N | R🡪IN4-N | R🡪P-N |
| 298.15 | 6.03E-18 | 2.97E-22 | 2.92E-22 | 2.99E-32 | 1.26E-32 |
| 300 | 9.17E-18 | 4.72E-22 | 4.64E-22 | 5.33E-32 | 2.20E-32 |
| 310 | 8.09E-17 | 5.30E-21 | 5.20E-21 | 1.10E-30 | 4.06E-31 |
| 320 | 6.27E-16 | 5.15E-20 | 5.05E-20 | 1.94E-29 | 6.47E-30 |
| 330 | 4.31E-15 | 4.39E-19 | 4.29E-19 | 2.91E-28 | 8.94E-29 |
| 340 | 2.66E-14 | 3.31E-18 | 3.23E-18 | 3.81E-27 | 1.08E-27 |
| 350 | 1.48E-13 | 2.23E-17 | 2.17E-17 | 4.37E-26 | 1.16E-26 |
| 360 | 7.53E-13 | 1.35E-16 | 1.32E-16 | 4.45E-25 | 1.10E-25 |
| **M06-2X/6-311+g(2df,2p)** | | | | | |
| T/K | R🡪IN1-N | R🡪IN2-N | R🡪IN3-N | R🡪IN4-N | R🡪P-N |
| 298.15 | 3.43E-18 | 1.32E-22 | 1.31E-22 | 1.48E-32 | 7.23E-33 |
| 300 | 5.23E-18 | 2.11E-22 | 2.08E-22 | 2.65E-32 | 1.27E-32 |
| 310 | 4.69E-17 | 2.36E-21 | 2.33E-21 | 5.60E-31 | 2.38E-31 |
| 320 | 3.68E-16 | 2.28E-20 | 2.25E-20 | 1.00E-29 | 3.87E-30 |
| 330 | 2.57E-15 | 1.94E-19 | 1.91E-19 | 1.55E-28 | 5.47E-29 |
| 340 | 1.60E-14 | 1.46E-18 | 1.44E-18 | 2.08E-27 | 6.80E-28 |
| 350 | 9.04E-14 | 9.85E-18 | 9.69E-18 | 2.44E-26 | 7.48E-27 |
| 360 | 4.65E-13 | 6.00E-17 | 5.89E-17 | 2.55E-25 | 7.32E-26 |

**Table S11.** Overall rate constants ( s^-1^) calculated by RRKM theory at the CBS-QB3, M06-2X/Jun-cc-pVTZ, and M06-2X/6-311+g(2df,2p) levels for all minimum stationary points of 3-hydroxyfuran-2(5H)-one unimolecular reaction in water.

| **CBS-QB3** | | | | | |
| --- | --- | --- | --- | --- | --- |
| T/K | R🡪IN1-O | R🡪IN2-O | R🡪IN3-O | R🡪IN4-O | R🡪P-O |
| 298.15 | 1.09E-25 | 1.19E-29 | 1.19E-29 | 6.92E-42 | 4.77E-37 |
| 300 | 1.87E-25 | 2.14E-29 | 2.13E-29 | 1.26E-41 | 9.36E-37 |
| 310 | 3.02E-24 | 4.41E-28 | 4.40E-28 | 3.71E-40 | 3.15E-35 |
| 320 | 4.14E-23 | 7.58E-27 | 7.56E-27 | 1.02E-38 | 8.56E-34 |
| 330 | 4.86E-22 | 1.10E-25 | 1.10E-25 | 2.37E-37 | 1.92E-32 |
| 340 | 4.96E-21 | 1.37E-24 | 1.37E-24 | 4.95E-36 | 3.60E-31 |
| 350 | 4.46E-20 | 1.49E-23 | 1.48E-23 | 9.08E-35 | 5.71E-30 |
| 360 | 3.55E-19 | 1.42E-22 | 1.41E-22 | 1.48E-33 | 7.80E-29 |
| **M06-2XJun-cc-pVTZ** | | | | | |
| T/K | R🡪IN1-O | R🡪IN2-O | R🡪IN3-O | R🡪IN4-O | R🡪P-O |
| 298.15 | 3.85E-24 | 2.33E-29 | 2.33E-29 | 8.63E-40 | 7.73E-38 |
| 300 | 6.45E-24 | 4.06E-29 | 4.06E-29 | 1.61E-39 | 1.46E-37 |
| 310 | 9.39E-23 | 7.39E-28 | 7.39E-28 | 4.11E-38 | 4.13E-36 |
| 320 | 1.16E-21 | 1.13E-26 | 1.13E-26 | 9.20E-37 | 9.80E-35 |
| 330 | 1.24E-20 | 1.49E-25 | 1.49E-25 | 1.82E-35 | 1.98E-33 |
| 340 | 1.15E-19 | 1.70E-24 | 1.70E-24 | 3.22E-34 | 3.44E-32 |
| 350 | 9.39E-19 | 1.70E-23 | 1.70E-23 | 5.09E-33 | 5.20E-31 |
| 360 | 6.86E-18 | 1.51E-22 | 1.50E-22 | 7.21E-32 | 6.86E-30 |
| **M06-2X/6-311+g(2df,2p)** | | | | | |
| T/K | R🡪IN1-O | R🡪IN2-O | R🡪IN3-O | R🡪IN4-O | R🡪P-O |
| 298.15 | 2.37E-24 | 2.31E-29 | 2.31E-29 | 8.77E-40 | 6.26E-37 |
| 300 | 3.98E-24 | 4.04E-29 | 4.04E-29 | 1.62E-39 | 1.19E-36 |
| 310 | 5.90E-23 | 7.40E-28 | 7.40E-28 | 4.01E-38 | 3.46E-35 |
| 320 | 7.40E-22 | 1.14E-26 | 1.14E-26 | 8.78E-37 | 8.35E-34 |
| 330 | 7.99E-21 | 1.51E-25 | 1.50E-25 | 1.71E-35 | 1.69E-32 |
| 340 | 7.51E-20 | 1.72E-24 | 1.72E-24 | 2.97E-34 | 2.93E-31 |
| 350 | 6.23E-19 | 1.72E-23 | 1.72E-23 | 4.64E-33 | 4.35E-30 |
| 360 | 4.61E-18 | 1.53E-22 | 1.53E-22 | 6.51E-32 | 5.63E-29 |

**Table S12.** Overall rate constants ( s^-1^) calculated by RRKM theory at the CBS-QB3, M06-2X/Jun-cc-pVTZ, and M06-2X/6-311+g(2df,2p) levels for all minimum stationary points of 3- hydroxythiophen-2(5H)-one unimolecular reaction in water.

| **CBS-QB3** | | | | | |
| --- | --- | --- | --- | --- | --- |
| T/K | R🡪IN1-S | R🡪IN2-S | R🡪IN3-S | R🡪IN4-S | R🡪P-S |
| 298.15 | 5.66E-22 | 4.04E-24 | 4.02E-24 | 5.13E-32 | 2.90E-32 |
| 300 | 9.13E-22 | 6.59E-24 | 6.55E-24 | 9.47E-32 | 5.22E-32 |
| 310 | 1.10E-20 | 8.36E-23 | 8.30E-23 | 2.31E-30 | 1.12E-30 |
| 320 | 1.15E-19 | 9.20E-22 | 9.14E-22 | 4.69E-29 | 2.03E-29 |
| 330 | 1.04E-18 | 8.77E-21 | 8.70E-21 | 8.01E-28 | 3.10E-28 |
| 340 | 8.29E-18 | 7.36E-20 | 7.30E-20 | 1.17E-26 | 4.07E-27 |
| 350 | 5.89E-17 | 5.51E-19 | 5.46E-19 | 1.47E-25 | 4.66E-26 |
| 360 | 3.77E-16 | 3.72E-18 | 3.68E-18 | 1.62E-24 | 4.69E-25 |
| **M06-2X/Jun-cc-pV(T+d)Z** | | | | | |
| T/K | R🡪IN1-S | R🡪IN2-S | R🡪IN3-S | R🡪IN4-S | R🡪P-S |
| 298.15 | 4.21E-21 | 2.88E-24 | 2.87E-24 | 1.77E-31 | 2.34E-32 |
| 300 | 6.72E-21 | 4.66E-24 | 4.65E-24 | 3.23E-31 | 4.10E-32 |
| 310 | 7.68E-20 | 5.75E-23 | 5.74E-23 | 7.44E-30 | 7.73E-31 |
| 320 | 7.56E-19 | 6.11E-22 | 6.10E-22 | 1.43E-28 | 1.25E-29 |
| 330 | 6.50E-18 | 5.68E-21 | 5.67E-21 | 2.32E-27 | 1.75E-28 |
| 340 | 4.93E-17 | 4.66E-20 | 4.65E-20 | 3.22E-26 | 2.13E-27 |
| 350 | 3.34E-16 | 3.42E-19 | 3.41E-19 | 3.89E-25 | 2.30E-26 |
| 360 | 2.04E-15 | 2.26E-18 | 2.26E-18 | 4.11E-24 | 2.21E-25 |
| **M06-2X/6-311+g(2df,2p)** | | | | | |
| T/K | R🡪IN1-S | R🡪IN2-S | R🡪IN3-S | R🡪IN4-S | R🡪P-S |
| 298.15 | 1.65E-21 | 1.12E-24 | 1.12E-24 | 4.53E-32 | 1.20E-32 |
| 300 | 2.66E-21 | 1.83E-24 | 1.83E-24 | 8.34E-32 | 2.13E-32 |
| 310 | 3.14E-20 | 2.33E-23 | 2.33E-23 | 2.00E-30 | 4.09E-31 |
| 320 | 3.18E-19 | 2.55E-22 | 2.54E-22 | 4.00E-29 | 6.76E-30 |
| 330 | 2.81E-18 | 2.44E-21 | 2.43E-21 | 6.74E-28 | 9.66E-29 |
| 340 | 2.19E-17 | 2.05E-20 | 2.05E-20 | 9.72E-27 | 1.20E-27 |
| 350 | 1.52E-16 | 1.54E-19 | 1.54E-19 | 1.21E-25 | 1.31E-26 |
| 360 | 9.51E-16 | 1.04E-18 | 1.04E-18 | 1.32E-24 | 1.28E-25 |

**Table S13.** Unscaled frequencies for of all stationary points of 3-hydroxyl-1H-pyrrol-2(5H)-one unimolecular reaction computed at the CBS-QB3 method in water.

| R-N | 109,253,300,335,421,466,545,620,774,789,805,810,954,1001,1050,1138,1209,1221,1263,1320,1415,1435,1485,1675,1699,3040,3066,3230,3623,3769 |
| --- | --- |
| IN1-N | 166,202,258,284,361,463,598,617,679,707,844,888,909,943,984,1096,1123,1150,1240,1323,1354,1406,1422,1556,1711,3018,3040,3258,3537,3759 |
| IN2-N | 122,216,276,307,478,509,564,656,705,748,808,902,950,996,1044,1078,1135,1205,1237,1311,1374,1381,1421,1658,1726,2996,3239,3259,3598,3780 |
| IN3-N | 68i,141,198,222,298,383,423,519,590,742,781,844,912,1040,1058,1123,1184,1255,1296,1386,1443,1472,1642,1711,1887,3065,3073,3159,3468,3771 |
| IN4-N | 149,212,265,298,439,449,516,577,630,664,844,889,893,949,970,1107,1150,1221,1233,1269,1326,1452,1540,1595,1738,3221,3281,3420,3473,3767 |
| P-N | 220,252,269,303,322,365,482,629,637,654,687,749,822,909,1065,1073,1143,1182,1267,1280,1393,1407,1500,1545,1657,3235,3272,3633,3782,3782 |
| TS1-N | 1166i,213,243,250,314,506,565,632,653,722,753,770,880,980,1025,1083,1135,1181,1217,1286,1383,1410,1465,1528,1641,2082,3229,3281,3597,3751 |
| TS2-N | 1191i,248,249,321,351,496,580,611,652,682,721,759,863,896,1051,1058,1134,1176,1206,1283,1351,1401,1446,1512,1628,2213,3250,3287,3585,3747 |
| TS3-N | 996i,249,277,350,395,469,512,641,652,722,757,862,895,926,1063,1097,1131,1182,1211,1300,1357,1427,1477,1534,1574,1858,3245,3260,3632,3756 |
| TS4-N | 1707i,226,265,337,396,442,489,604,638,698,737,757,810,870,888,1056,1088,1135,1234,1278,1354,1448,1478,1515,1606,2530,3242,3262,3622,3794 |
| TS5-N | 1714i,193,228,293,349,505,539,588,635,678,735,768,847,872,938,1027,1111,1190,1216,1241,1339,1382,1452,1539,1646,2199,3222,3264,3554,3788 |
| TS6-N | 1522i,236,244,288,354,443,554,629,635,673,694,780,855,868,926,1074,1094,1151,1239,1257,1354,1387,1483,1496,1627,1804,3232,3256,3566,3780 |

**Table S14.** Unscaled frequencies for of all stationary points of 3-hydroxyfuran-2(5H)-one unimolecular reaction computed at the CBS-QB3 method in water.

| R-O | 189,262,304,317,451,554,616,762,786,802,818,946,1015,1045,1066,1195,1203,1236,1374,1426,1472,1690,1754,3064,3105,3236,3765 |
| --- | --- |
| IN1-O | 44,149,170,217,218,312,341,432,619,725,756,824,960,1051,1203,1217,1334,1394,1398,1433,1458,1744,2154,2989,3007,3022,3796 |
| IN2-O | 116,241,306,320,507,555,635,725,760,809,880,942,980,1064,1106,1129,1207,1214,1307,1365,1430,1688,1820,3026,3247,3273,3791 |
| IN3-O | 68,140,198,230,293,383,475,551,601,782,848,860,1036,1042,1057,1204,1291,1386,1434,1453,1594,1706,1733,2970,3081,3169,3754 |
| IN4-O | 50,132,199,227,264,291,352,474,553,627,722,749,826,900,1024,1205,1255,1270,1306,1412,1446,1709,2135,3191,3210,3748,3805 |
| P-O | 175,261,276,286,322,489,611,637,646,699,746,846,891,1042,1052,1130,1170,1266,1297,1345,1453,1543,1696,3244,3292,3775,3780 |
| TS1-O | 1021i,133,231,273,312,490,588,596,684,710,752,878,908,979,1044,1168,1207,1240,1300,1379,1438,1507,1727,2147,3221,3260,3768 |
| TS2-O | 1076i,228,252,300,344,515,571,601,641,694,759,856,904,1018,1040,1118,1154,1203,1289,1348,1435,1506,1683,2309,3246,3291,3751 |
| TS3-O | 1022i,250,289,385,478,513,617,634,721,754,858,899,922,961,1071,1152,1181,1212,1306,1391,1462,1576,1637,1841,3248,3268,3741 |
| TS4-O | 1730i,225,271,336,431,488,560,627,689,731,776,807,877,883,1053,1080,1132,1240,1286,1309,1450,1512,1598,2517,3250,3271,3776 |
| TS5-O | 1667i,201,242,307,347,490,509,557,596,694,709,746,819,898,933,1079,1133,1195,1222,1335,1459,1557,1745,2343,3215,3228,3791 |
| TS6-O | 1320i,137,160,250,337,409,480,544,633,690,780,800,889,950,1076,1110,1162,1245,1300,1419,1484,1572,1685,1765,3164,3205,3715 |

**Table S15.** Unscaled frequencies for of all stationary points of 3- hydroxythiophen-2(5H)-one unimolecular reaction computed at the CBS-QB3 method in water.

| R-S | 136,250,298,347,424,427,480,592,681,708,748,804,913,937,1024,1138,1141,1225,1269,1396,1439,1669,1691,3062,3103,3202,3775 |
| --- | --- |
| IN1-S | 153,220,272,275,322,382,451,561,585,648,722,809,860,919,1005,1079,1161,1223,1285,1300,1329,1447,1621,3000,3016,3248,3753 |
| IN2-S | 81,222,284,297,406,470,514,581,664,690,746,808,935,949,1036,1113,1163,1218,1303,1322,1425,1646,1738,2984,3205,3229,3778 |
| IN3-S | 38,80,247,251,323,346,482,540,613,683,814,851,907,940,982,1136,1214,1294,1373,1407,1445,1579,1713,3039,3122,3152,3737 |
| IN4-S | 62,100,181,197,246,280,328,463,514,584,677,703,734,829,930,963,1193,1266,1308,1392,1410,1640,2118,2611,3151,3201,3753 |
| P-S | 178,195,240,250,292,416,483,520,583,641,685,717,821,860,960,1088,1118,1243,1281,1338,1423,1488,1641,3202,3258,3760,3803 |
| TS1-S | 1047i,180,264,302,310,367,479,496,609,621,687,746,859,917,950,1058,1173,1193,1258,1285,1392,1520,1608,2242,3196,3261,3747 |
| TS2-S | 1091i,189,261,333,400,412,478,496,542,617,648,740,841,889,940,1069,1142,1177,1257,1310,1423,1477,1569,2209,3218,3268,3730 |
| TS3-S | 1091i,189,261,333,400,412,478,496,542,617,648,740,841,889,940,1069,1142,1177,1257,1310,1423,1477,1569,2209,3218,3268,3730 |
| TS4-S | 1658i,180,269,310,394,419,470,507,625,650,694,718,775,832,894,949,1099,1153,1268,1305,1433,1468,1567,2550,3213,3241,3770 |
| TS5-S | 1238i,168,273,287,317,343,455,463,528,598,643,672,769,875,883,903,1083,1187,1239,1290,1470,1545,1642,2029,3202,3280,3757 |
| TS6-S | 1037i,102,172,288,314,375,445,492,600,630,709,751,793,866,919,987,1151,1197,1247,1374,1408,1483,1571,1872,3175,3205,3749 |

**Table S16.** Cartesian coordinates of all components of 3-hydroxyl-1H-pyrrol-2(5H)-one unimolecular, 3-hydroxyfuran-2(5H)-one, and 3-hydroxythiophen-2(5H)-one reactions calculated at the M06-2X/Jun-cc-pVTZ level in water.

R-N

C 0.13105900 0.84755100 0.00044200

C 0.58455700 -0.56698100 0.00000600

C -0.46930700 -1.37940400 0.00007600

C -1.71852700 -0.55862000 -0.00003800

N -1.20998300 0.79974800 -0.00007100

O 0.84966400 1.84815900 -0.00004100

O 1.91715200 -0.79516100 -0.00016700

H 2.08863000 -1.74603000 -0.00035200

H -2.33075400 -0.74863600 -0.88280800

H -1.79067800 1.62370100 -0.00021200

H -0.46762700 -2.45800000 -0.00002500

H -2.33091600 -0.74852500 0.88264300

IN1-N

C 0.28361500 0.78129700 -0.00010900

C 0.52991900 -0.55041500 -0.00010000

C -0.72387200 -1.34006500 0.00010400

C -1.75628300 -0.28647000 0.00014000

N -1.18195600 0.86164000 -0.00005600

O 0.99274900 1.84120600 -0.00013700

O 1.76361200 -1.16176400 -0.00010200

H 2.44274400 -0.47420900 0.00151400

H -2.82793000 -0.40888400 0.00016500

H -1.67118100 1.75277100 -0.00014200

H -0.86069500 -1.98158400 -0.87755500

H -0.86040900 -1.98119100 0.87810600

IN2-N

C 0.26445500 0.82024500 0.09451700

C 0.47492500 -0.67214900 0.38936200

C -0.88444500 -1.25200200 0.14069400

C -1.72312800 -0.25455600 -0.11945900

N -1.05799800 0.99004500 -0.12828400

O 1.13621400 1.67514900 0.05897500

O 1.48148000 -1.23230600 -0.42914200

H 2.33035600 -0.85961000 -0.16056600

H -2.78559400 -0.27727800 -0.29783800

H -1.47933500 1.86792500 -0.39492800

H -1.13644800 -2.29467800 0.23393300

H 0.74460800 -0.75864000 1.44804000

IN3-N

C 0.79445300 -0.94146100 0.20825300

C 0.51999300 0.50201200 0.01863600

C -0.70905100 1.03172500 0.08129000

C -1.95234100 0.27192300 0.19585000

N -2.14826200 -0.84242600 -0.39191800

O 1.88735400 -1.41842000 -0.00822800

O 1.65328000 1.22276900 -0.15221100

H 1.43624000 2.15554600 -0.28497700

H -2.72633000 0.74823100 0.79794200

H -3.07682400 -1.19274600 -0.15710000

H -0.80664000 2.11208800 0.05794800

H -0.03200800 -1.54612300 0.58895500

IN4-N

C 1.12033300 0.73384400 0.13691900

C 0.68663600 -0.50554200 0.08210100

C -0.60203300 -0.84647200 0.68751100

C -1.77551800 -0.29272800 0.32515700

N -1.99271600 0.62143500 -0.66176000

O 1.45044300 1.85017800 0.22193100

O 1.51179600 -1.48245300 -0.48639100

H 1.03962800 -1.86408800 -1.23804100

H -2.67650200 -0.60966600 0.83535800

H -2.87160500 1.11033500 -0.66338800

H -0.60480200 -1.58687200 1.47710400

H -1.21213200 1.12384200 -1.05315900

P-N

C -0.20249400 0.69467800 0.00357900

C -0.41940900 -0.65560800 -0.01326300

C 0.85536400 -1.28972700 -0.01569100

C 1.79490100 -0.29382900 0.00543500

N 1.13843500 0.91424800 0.01056200

O -1.05691400 1.75260800 0.00181600

O -1.67522800 -1.23692200 -0.08453200

H -1.80001800 -1.81341400 0.67821000

H 2.86905800 -0.33617400 0.01635800

H 1.56834300 1.82603900 0.01999000

H 1.04293400 -2.35051100 -0.02667800

H -1.96239400 1.41575500 0.01956700

TS1-N

C 0.25971500 0.77704000 -0.03553400

C 0.51010500 -0.59054300 -0.04846100

C -0.71519700 -1.29676000 -0.02632600

C -1.75010500 -0.29882100 -0.03939700

N -1.14360100 0.89382600 -0.02560200

O 1.04476800 1.76875300 0.01910000

O 1.75587600 -1.17269800 -0.00570300

H 2.40263100 -0.47525800 0.16621300

H -2.81332600 -0.44299000 -0.11728400

H -1.61705000 1.77831700 0.10188200

H -0.88966800 -2.34079500 -0.23507900

H -1.30964100 -1.08999100 1.05460700

TS2-N

C 0.20970800 0.81044100 -0.00271900

C 0.53187900 -0.55981300 -0.00726800

C -0.73545500 -1.33262900 0.00500000

C -1.75151200 -0.38144800 -0.04524200

N -1.17402900 0.83878800 -0.02720400

O 0.96167700 1.83190800 0.00768900

O 1.76591300 -1.11675400 -0.10388100

H 2.40965800 -0.53774300 0.32786600

H -2.81767300 -0.51371100 -0.05096300

H -1.68999100 1.70822300 0.02788800

H -0.81679000 -2.39771700 -0.12908800

H -0.21544600 -1.07110000 1.08563000

TS3-N

C 0.06510400 0.83974500 0.09037100

C 0.60287800 -0.51365400 0.00908100

C -0.46327700 -1.44089200 -0.05031000

C -1.61322600 -0.69555400 -0.05689200

N -1.33533300 0.61896700 0.00413200

O 0.63184900 1.96828200 -0.11065600

O 1.91866400 -0.71227800 -0.05786400

H 2.13711000 -1.62632400 0.17291200

H -2.63383300 -1.04204600 -0.03884600

H -2.01288100 1.36523200 0.05280100

H -0.38042000 -2.51330300 -0.04008300

H 0.28438200 0.29777400 1.21895000

TS4-N

C 0.19002600 0.74460200 0.08018200

C 0.46818200 -0.63976100 0.00651700

C -0.74183100 -1.33876500 -0.01408200

C -1.74182800 -0.37985500 -0.02942300

N -1.20279800 0.84839100 -0.01934600

O 1.05921800 1.82683300 -0.12773400

O 1.72979100 -1.10171100 -0.02717700

H 1.73060800 -2.06403300 0.05758500

H -2.81011900 -0.51630600 -0.00490700

H -1.71084700 1.71841800 0.03591800

H -0.87620500 -2.40676700 -0.01079900

H 0.72678600 1.21164700 1.03775100

TS5-N

C 0.25993100 0.77008400 -0.04133800

C 0.50136600 -0.61180400 -0.03651200

C -0.68560800 -1.33191300 -0.01181900

C -1.75690800 -0.46019000 -0.00388500

N -1.14196700 0.88929700 0.00034900

O 1.05640900 1.74365200 0.00379100

O 1.75998700 -1.14651600 0.01296300

H 2.39330100 -0.41594100 0.04943700

H -2.78076700 -0.55210300 -0.31629800

H -1.64318100 1.70693600 -0.33423900

H -0.77331200 -2.40655700 0.01686600

H -1.64612000 0.46843600 1.00909100

TS6-N

C 0.12891900 0.83049700 0.04562400

C 0.61647900 -0.49285400 0.03079500

C -0.42420100 -1.41747800 -0.09541000

C -1.62251300 -0.74196000 -0.09343100

N -1.35800600 0.58648500 0.06419800

O 0.64003800 1.96592300 -0.14828300

O 1.95803200 -0.71032000 0.03271700

H 2.13633100 -1.65486300 0.12180900

H -2.62623600 -1.11884700 -0.00339800

H -2.01852400 1.35417400 -0.00164400

H -0.30799900 -2.48887200 -0.11932100

H -0.65419100 0.68895200 1.15223200

R-O

C -0.11912700 0.83607400 0.00010200

C -0.58841200 -0.56150900 0.00002300

C 0.46662600 -1.36826500 0.00003200

C 1.69807800 -0.53098000 -0.00000500

O -0.77096000 1.85280200 -0.00008800

O -1.91984000 -0.77185400 -0.00000800

H -2.10579900 -1.72031300 -0.00002700

H 2.31165400 -0.67616300 0.88866200

H 0.47731500 -2.44680800 0.00002000

H 2.31159800 -0.67618900 -0.88870600

O 1.22358100 0.82749700 -0.00001200

IN1-O

C 0.34819400 0.79711800 -0.00009400

C 0.54120600 -0.52987900 -0.00004100

C -0.70786500 -1.32046000 -0.00002200

C -1.72827000 -0.27093400 -0.00012300

O 0.91577200 1.88537500 -0.00006100

O 1.76825500 -1.16912800 -0.00006500

H 2.46941100 -0.50542300 0.00034800

H -2.80423600 -0.41557300 0.00012000

H -0.86298400 -1.96147700 -0.87713500

H -0.86300200 -1.96147800 0.87709000

O -1.26637500 0.88236200 0.00028200

IN2-O

C 0.24703100 0.81815000 0.09528100

C 0.49103400 -0.66115400 0.40188200

C -0.86418100 -1.23978600 0.15554900

C -1.69145400 -0.25109200 -0.12847600

O 1.04020900 1.71294500 0.05250000

O 1.48985800 -1.20131700 -0.43583700

H 2.34989600 -0.90509500 -0.11327400

H -2.74870600 -0.22848900 -0.33299900

H -1.12287100 -2.28067900 0.24941400

H 0.77733000 -0.74240000 1.45474700

O -1.07384600 1.00836600 -0.16707700

IN3-O

C 0.82680300 -0.95066500 -0.00000500

C 0.48683700 0.50500200 -0.00006200

C -0.76794600 0.99282600 0.00003300

C -2.01777700 0.23406200 0.00018000

O 1.97732800 -1.31829200 -0.00004700

O 1.58301600 1.27161100 -0.00021100

H 1.34587400 2.21012300 -0.00026500

H -2.91299600 0.87132800 0.00033400

H -0.89023300 2.06964600 0.00001300

H -0.01172300 -1.64910400 0.00033300

O -2.14764800 -0.97698600 0.00009700

IN4-N

C -0.80290900 0.88581100 -0.03225400

C -0.72440100 -0.43549900 -0.03110100

C 0.50763300 -1.20447800 -0.10887100

C 1.73080000 -0.68911700 -0.03480800

O -0.93672700 2.03969800 -0.05728100

O -1.95331400 -1.10456600 0.00374500

H -2.09543200 -1.43541600 0.89999300

H 2.63110600 -1.28407600 -0.09152200

H 0.38669500 -2.27082800 -0.23541700

H 2.82383900 0.88013200 0.18928600

O 1.88842200 0.66110300 0.11351900

P-O

C -0.22932000 0.68038500 0.00765700

C -0.40318000 -0.65929000 -0.00567300

C 0.91128300 -1.23364700 -0.01155500

C 1.77386800 -0.19786500 0.00120400

O -1.05651300 1.74138700 0.00292200

O -1.62322000 -1.29133300 -0.06158400

H -1.57799400 -2.10791300 0.45005100

H 2.84533500 -0.11701600 0.00994300

H 1.15719700 -2.28233800 -0.01847000

H -1.97084600 1.42823100 0.03571100

O 1.08353400 0.99263800 0.00528400

TS1-O

C 0.24336500 0.79554200 -0.03150800

C 0.54904700 -0.54622400 -0.05063000

C -0.66598200 -1.30461700 -0.02545000

C -1.70366200 -0.34299500 -0.04985900

O 0.87066300 1.86093900 0.00551800

O 1.81897400 -1.08029700 -0.07176300

H 2.32573000 -0.72836600 0.67133200

H -2.77406500 -0.47734800 -0.07098000

H -0.80691600 -2.34645800 -0.27474000

H -1.19418800 -1.19496600 1.06938700

O -1.20053300 0.86147000 0.00995400

TS2-O

C 0.21985900 0.80830100 -0.00872800

C 0.53707100 -0.55026300 -0.03180400

C -0.74019500 -1.30963900 -0.00117300

C -1.72954400 -0.34243400 -0.04068100

O 0.89460600 1.85013000 0.00283700

O 1.76311700 -1.11842000 -0.09771300

H 2.39280500 -0.59827900 0.42069300

H -2.80253900 -0.41015200 -0.06737600

H -0.85476000 -2.37074300 -0.14716700

H -0.29894100 -1.06618800 1.08855000

O -1.17768600 0.86948600 -0.00517200

TS3-O

C 0.10085500 0.83521300 0.09162900

C 0.58523400 -0.52244400 0.00313800

C -0.53341800 -1.40708500 -0.05143900

C -1.61742200 -0.60635300 -0.05401100

O 0.63937500 1.94984900 -0.11433900

O 1.87676000 -0.78257500 -0.05690800

H 2.05876400 -1.70713100 0.17032100

H -2.66757800 -0.85150800 -0.04388900

H -0.50384900 -2.48228400 -0.05725100

H 0.29201300 0.29696300 1.23351200

O -1.31499100 0.70122200 0.01642300

TS4-O

C 0.25736000 0.72112200 0.07757700

C 0.41402400 -0.66728600 -0.00270700

C -0.87553200 -1.24735600 -0.01073700

C -1.73935600 -0.19992500 -0.02537000

O 1.15458200 1.74928600 -0.12320900

O 1.61066200 -1.24880300 -0.03306200

H 1.52649600 -2.20576800 0.07839500

H -2.81626500 -0.16618200 -0.01107300

H -1.12226600 -2.29501400 -0.00853300

H 0.83060000 1.12236900 1.04795700

O -1.10993700 0.98767500 -0.01114400

TS5-O

C 0.14651400 0.82416100 -0.04708300

C 0.60396800 -0.50166300 -0.01871800

C -0.45305800 -1.38755400 0.03801300

C -1.65765300 -0.71505200 0.04039100

O 0.67478100 1.92535700 0.02575300

O 1.94830400 -0.74816100 0.01966900

H 2.11205600 -1.67690000 -0.18592600

H -2.59103400 -0.93135000 -0.45125400

H -0.36948000 -2.46370100 0.07365800

H -1.75852400 0.44507800 0.87564000

O -1.27704100 0.73624500 -0.09389000

TS6-O

C 0.71165700 0.86579500 0.16424900

C 0.55601600 -0.55357000 -0.10059200

C -0.72095300 -1.09170300 -0.30120300

C -1.83185400 -0.31578600 -0.06623100

O 1.51736700 1.66773800 -0.23703300

O 1.60759200 -1.30401600 0.21376500

H 1.34909700 -2.22394900 0.38248500

H -2.80863100 -0.78101300 0.02316600

H -0.83360400 -2.15896900 -0.42760000

H -0.72963800 1.16575100 0.51772500

O -1.78326100 0.95749800 0.18912800

R-S

C -0.22810400 0.84051200 -0.00002400

C -0.94238600 -0.45330600 0.00004800

C -0.15443300 -1.52511100 -0.00004500

C 1.30734300 -1.24763600 -0.00000400

O -0.75535300 1.93292500 0.00000800

O -2.29289700 -0.36117900 0.00006600

H -2.68544700 -1.24427800 0.00004400

H 1.79695200 -1.65856200 0.88262900

H -0.51617500 -2.54408000 0.00000900

H 1.79701500 -1.65864500 -0.88255900

S 1.50619600 0.55280300 -0.00003600

IN1-S

C 0.36443600 0.80539500 -0.00016300

C 0.86348600 -0.46282300 -0.00039300

C -0.09816100 -1.57451300 -0.00011600

C -1.45121000 -1.01308000 0.00025700

O 0.90759000 1.94770700 0.00003100

O 2.20413000 -0.73931000 0.00011800

H 2.68846600 0.09830500 0.00074100

H -2.35895200 -1.59845900 0.00005200

H 0.00177100 -2.23850500 -0.87063200

H 0.00297400 -2.23828500 0.87067400

S -1.45620800 0.61124300 0.00002900

IN2-S

C 0.16589400 0.84846700 0.10714600

C 0.98262300 -0.42396900 0.37170900

C 0.08089200 -1.58348800 0.12317400

C -1.19121300 -1.27020900 -0.07416400

O 0.63042200 1.95542300 0.04023900

O 2.14603500 -0.46316900 -0.42237000

H 2.75730900 0.21325300 -0.10529700

H -2.00941200 -1.95880700 -0.22214000

H 0.45022700 -2.59797500 0.16072400

H 1.23920000 -0.38124300 1.43875000

S -1.55488500 0.46012100 -0.08638600

IN3-S

C -1.03503400 -1.01392700 -0.00048300

C -0.98842400 0.47753900 -0.00014900

C 0.13419500 1.23388400 -0.00044700

C 1.52909400 0.86778600 -0.00076200

O -2.08976300 -1.60342200 -0.00008700

O -2.21748700 1.00554800 0.00025500

H -2.18248600 1.97317800 0.00045100

H 2.16209400 1.75271500 -0.00088100

H -0.03143200 2.30669700 -0.00045600

H -0.07554100 -1.53494800 -0.00142600

S 2.29664800 -0.56914700 0.00075000

IN4-S

C -1.01917400 0.97789600 -0.15252500

C -1.17476700 -0.32922300 -0.02712300

C -0.14718900 -1.27176900 -0.44099100

C 1.17485600 -1.10648400 -0.35234600

O -0.87888400 2.11791600 -0.31660700

O -2.40966400 -0.79024500 0.42626300

H -2.27779800 -1.21088900 1.28609500

H 1.84213400 -1.86724300 -0.73174900

H -0.52689200 -2.19522200 -0.86472200

H 3.16017200 -0.21417500 0.44001300

S 1.94427500 0.32772800 0.30193900

P-S

C -0.23165200 0.70180400 -0.00004200

C -0.83542600 -0.51571500 -0.00002200

C 0.09871500 -1.59005300 0.00006000

C 1.38480600 -1.15622700 -0.00001100

O -0.80734300 1.93866400 -0.00018600

O -2.20481500 -0.62721500 0.00026000

H -2.44991400 -1.56011800 -0.00190500

H 2.28864200 -1.74224400 0.00001600

H -0.19559700 -2.63004900 0.00007700

H -1.76903100 1.83556500 0.00103300

S 1.48278100 0.56040000 0.00001700

TS1-S

C 0.34013000 0.79288500 -0.02249600

C 0.87155200 -0.48433600 -0.02756100

C -0.07764600 -1.54413000 -0.02439600

C -1.42194800 -1.05076800 -0.05787800

O 0.91508000 1.90933400 0.01147000

O 2.22162300 -0.72713900 -0.00676700

H 2.67649400 0.12388500 0.06671900

H -2.30589200 -1.66664400 -0.11182600

H 0.15573200 -2.56743700 -0.28888400

H -0.59250000 -1.63086800 1.06227900

S -1.45624900 0.62510000 0.00175400

TS2-S

C 0.29040400 0.81306800 0.00849600

C 0.87190700 -0.47348100 -0.02305300

C -0.10312400 -1.56745700 0.01197700

C -1.41533700 -1.09341100 -0.04347900

O 0.88098500 1.91943700 0.01515700

O 2.20044300 -0.69452500 -0.09476200

H 2.66767700 0.09673500 0.21451700

H -2.31559200 -1.68306700 -0.05668600

H 0.18783300 -2.59914900 -0.11815500

H 0.25724300 -1.19627900 1.10643800

S -1.45698000 0.59438500 -0.01455700

TS3-S

C 0.21430000 0.84605900 0.08608100

C 0.92794200 -0.41075800 0.00762600

C 0.09808000 -1.57119900 -0.02554200

C -1.21605300 -1.25734000 -0.05049500

O 0.65760300 2.01812600 -0.09011400

O 2.24946700 -0.37716500 -0.06412900

H 2.63472200 -1.24937400 0.10945100

H -2.03109600 -1.96539600 -0.01972500

H 0.49718900 -2.57357500 -0.00155900

H 0.41554900 0.32067700 1.22316500

S -1.55740900 0.41871400 -0.01146300

TS4-S

C 0.22234600 0.75785200 0.06907100

C 0.88207000 -0.48283600 0.00051000

C 0.00777500 -1.58913100 0.01642700

C -1.29868600 -1.20223400 -0.00298400

O 0.81417900 2.01233900 -0.08169100

O 2.21448700 -0.51971700 -0.05686400

H 2.53221200 -1.43095100 0.01203400

H -2.15883800 -1.85293900 0.04180500

H 0.34243000 -2.61529900 0.04286600

H 0.56087500 1.28193200 1.07839300

S -1.52419000 0.48589800 -0.03530000

TS5-S

C 0.36066800 0.78456900 -0.01444100

C 0.86956600 -0.50817800 -0.03479700

C -0.05760700 -1.56095900 -0.00647400

C -1.36304300 -1.18354100 -0.01267700

O 0.93244600 1.89435000 0.03601000

O 2.21921300 -0.73427300 -0.00081000

H 2.67225600 0.11958800 0.04503900

H -2.27976700 -1.74402200 -0.06241400

H 0.24359900 -2.59875800 0.03130300

H -1.65828400 -0.11133000 1.15826700

S -1.44053600 0.61641000 -0.06521600

TS6-S

C 0.40227800 0.80497000 0.01416900

C 0.87799100 -0.51212700 0.03878200

C -0.06344400 -1.54349200 -0.03701400

C -1.36897000 -1.14431500 -0.07305800

O 0.93197100 1.91174600 -0.09589500

O 2.21722400 -0.75631900 0.01467700

H 2.69714600 0.08390100 0.01083000

H -2.24442000 -1.76709500 0.00997100

H 0.23295000 -2.58265100 -0.02615700

H -0.55478100 0.68925400 1.15724500

S -1.52572400 0.54393500 -0.00996400
